# Test-retest reliability of the play-or-pass version of the Iowa Gambling Task

**DOI:** 10.3758/s13415-024-01197-6

**Published:** 2024-06-07

**Authors:** Jeremy M. Haynes, Nathaniel Haines, Holly Sullivan-Toole, Thomas M. Olino

**Affiliations:** 1 Department of Psychology and Neuroscience, Temple University, 1701 N. 13th Street, Philadelphia, PA 19122 USA; 2 Bayesian Beginnings LLC, Columbus, Ohio USA

**Keywords:** Iowa Gambling Task, Test-retest reliability, Construct validity, Computational modeling, Hierarchical Bayesian analysis

## Abstract

The Iowa Gambling Task (IGT) is used to assess decision-making in clinical populations. The original IGT does not disambiguate reward and punishment learning; however, an adaptation of the task, the “play-or-pass” IGT, was developed to better distinguish between reward and punishment learning. We evaluated the test-retest reliability of measures of reward and punishment learning from the play-or-pass IGT and examined associations with self-reported measures of reward/punishment sensitivity and internalizing symptoms. Participants completed the task across two sessions, and we calculated mean-level differences and rank-order stability of behavioral measures across the two sessions using traditional scoring, involving session-wide choice proportions, and computational modeling, involving estimates of different aspects of trial-level learning. Measures using both approaches were reliable; however, computational modeling provided more insights regarding between-session changes in performance, and how performance related to self-reported measures of reward/punishment sensitivity and internalizing symptoms. Our results show promise in using the play-or-pass IGT to assess decision-making; however, further work is still necessary to validate the play-or-pass IGT.

**Supplementary Information:**

The online version contains supplementary material available at 10.3758/s13415-024-01197-6.

The Iowa Gambling Task (IGT; Bechara et al., 1994) is frequently used to assess reward learning within the Positive Valence Systems of the Research Domain Criteria (PVS Work Group, 2011). During the task, participants make choices between one of four decks of cards, two of which have advantageous outcomes (i.e., net monetary wins) and two of which have disadvantageous outcomes (i.e., net monetary losses), on average, across trials. Based on trial-by-trial feedback, participants can learn to select advantageous decks and avoid disadvantageous decks. Learning within the task is thought to reflect aspects of affective processing such that “somatic markers” (i.e., the emotional signals associated with wins & losses) guide individuals to choose the advantageous decks and avoid the disadvantageous decks. The IGT was initially developed to identify decision-making deficits among individuals with damage to the ventromedial prefrontal cortex (VMPFC; Bechara et al., 1994, 1997), an area of the brain implicated in emotion regulation (Winecoff et al., 2013). Subsequent studies have extended these findings by showing that impairments in other areas of the prefrontal cortex (e.g., medial PFC), as well as regions outside of the prefrontal cortex (e.g., amygdala), also are related to decision-making deficits on the IGT (Aram et al., 2019). Based on these relations between IGT performance and neurological functioning, use of the IGT has been extended to other clinical populations (Bechara, 2007; Buelow & Suhr, 2009), including depression (Cella et al., 2010; Must et al., 2006; Siqueira et al., 2018) and substance use (Solowij et al., 2012). Despite the popularity of its use, the test-retest reliability of IGT performance (Buelow & Barnhart, 2018; Schmitz et al., 2020; Sullivan-Toole et al., 2022), and associations between IGT performance and individual difference characteristics, vary considerably across studies (Baeza-Velasco et al., 2020; Byrne et al., 2016; Case & Olino, 2020; Jollant, 2016; McGovern et al., 2014; Mueller et al., 2010; Smoski et al., 2008). Thus, there are outstanding concerns regarding the reliability and validity of the IGT that must be addressed for it to be used as a clinical assessment tool.

We focused on two aspects of the IGT that could influence the reliability and validity of the IGT: how the task is structured, and how behavioral performance is quantified. First, the original IGT is structured such that participants can choose among the four decks simultaneously and choices between the different decks are mutually exclusive. Therefore, participants’ performance conflates approach toward the advantageous decks, reflecting reward learning, and avoidance of the disadvantageous decks, reflecting punishment learning. Given the variability in performance on the task, later studies altered the IGT such that participants are presented with a card from a specific deck on each trial, and they have the opportunity to “play” or “pass” on that card (Peters & Slovic, 2000). Thus, in the revised play-or-pass version of the IGT, reward and punishment learning are dissociable. This is an attractive feature of the play-or-pass IGT because previous research indicates that there are both behavioral and neuropsychological differences in reward and punishment learning, which could be more readily captured by this version of the IGT (Christakou, et al., 2013; Frank et al., 2004; Gershman, 2015). This revised version of the IGT shows some promise with there being expected associations between task performance and pubertal development (Icenogle et al., 2017); however, there are inconsistent associations with clinical outcomes (Case & Olino, 2020). To our knowledge, the reliability of the play-or-pass IGT has yet to be tested; thus, a major goal of this study was to assess the reliability of measures from this version of the IGT.

The second aspect of the IGT that could influence reliability and validity is how behavior is characterized on the task. Traditionally, the proportion of selections for “good” (i.e., advantageous) and “bad” (i.e., disadvantageous) decks have been used as measures of reward and punishment learning, respectively; however, such measures are gross characterizations of behavior, which can be problematic because they do not capture learning *within* the task. Some studies have employed changes in choice proportions in early versus late trials (or blocks) as measures of within-session learning (Brand et al., 2007); however, these approaches do not capture the specific processes that may influence trial-by-trial learning within the task. An alternative approach to these summary measures comes from advancements in computational modeling. Computational models mathematically delineate the theoretically hypothesized processes that give rise to the observed data (i.e., choices within the task). This allows us to characterize individual differences in task performance that may otherwise be obscured by summary measures.

Given the issues associated with using summary measures of behavior, a second major goal of this study was to extend the Outcome-Representation Learning (ORL) model to this version of the IGT. The ORL model is a trial-level reinforcement learning computational model that was developed for the original IGT (Haines et al., 2018) that builds on previous computational models for the IGT (Ahn et al., 2008; Busemeyer & Stout, 2002; Worthy et al., 2013). The ORL model performs well in predicting participants’ earnings and trial-to-trial choices and decomposes task behavior into distinct processes, yielding five parameters: reward learning rate, punishment learning rate, win frequency sensitivity, perseveration tendency, and memory decay. These parameters inform a computation of the subjective value of each deck, which is updated on a trial-by-trial basis depending on the outcome received. Importantly, Haines et al. showed that individual differences in ORL parameters were related to socially significant individual differences that may be of interest to clinicians (e.g., substance use; see also Kildahl et al., 2020). More recently, Sullivan-Toole et al. (2022) found that individual differences in ORL parameters were related to internalizing symptoms (e.g., depressive symptoms). Thus, parameters from a computational model, such as the ORL, may be useful to characterize facets of decision-making related to human health (e.g., depression).

In their study, Sullivan-Toole et al. showed that the most reliable ORL parameters were obtained by using joint modeling—an approach that involves fitting a model to data from multiple sessions to estimate parameters and their reliabilities within a single model (Haines et al., 2020). Joint modeling involves specifying a full probabilistic model of both within- and between-person variability, captured by behavioral (e.g., the ORL) and group-level (e.g., a multivariate normal distribution across ORL parameters) models. The joint probabilistic structure of the full model allows for information to flow from the person- to group-level parameters. Because person-level parameters are constrained by their respective group-level parameters, this flow of information has the effect of pooling person-level parameter estimates toward their group-level means (Brown et al., 2020). Also referred to as “regression toward the mean,” pooling reduces within-person variability attributable to measurement error that attenuates correlations (Matzke et al., 2017). In fact, there is a mathematical equivalence between classical test theory “true score estimates” (including disattenuated correlation estimates) and the parameter estimates derived from appropriately specified joint models (Haines et al., 2023). Beyond the theoretical correspondence, a number of studies have demonstrated that joint modeling improves reliability in a practical sense (Karvelis et al., 2023), and as a result it is an increasingly recommended approach for applied modeling of behavioral data in the social sciences (Haines et al., 2023; Zorowitz & Niv, 2023). The joint ORL model introduced by Sullivan-Toole et al. relied on the original IGT design and has not been applied to the play-or-pass version of the IGT.

Thus, in this study, we tested participants on the play-or-pass IGT across two sessions and extended the ORL model to this version of the task to address the following three goals. First, we evaluated the test-retest reliability of performance on the play-or-pass IGT by using a traditional scoring and joint computational modeling approach. Second, we evaluated the degree to which reward and punishment learning were dissociable using the traditional scoring and computational modeling approaches. The task-structure of the play-or-pass IGT permits clearer separation between reward and punishment learning using traditional scoring procedures (Case & Olino, 2020; Cauffman et al., 2010; Peters & Slovic, 2000); however, to our knowledge, this distinction has not been directly examined. In addition, although measures of reward and punishment learning from the ORL model do not depend on the task structure per se,^1^ it is still important to determine whether the model could distinguish reward and punishment learning as in the traditional scoring approach. Finally, given inconsistencies in the associations between IGT performance and purported correlates, including reward sensitivity, threat sensitivity, personality, and internalizing symptoms (Buelow & Suhr, 2009; Case & Olino, 2020), we examined associations between IGT performance and these characteristics.

## Method

### Participants and procedure

Participants were 49 (39 females) undergraduate students, who attended a large university in the United States and were recruited by using SONA—an online research management platform. They received course credit for participation and a monetary bonus based on performance in the IGT. This sample size was chosen because it is within the range of what our lab has successfully used to examine test-retest reliability for the original IGT using a joint computational modeling approach (*N* = 50; Sullivan-Toole et al., 2022). Participant ages ranged from 18 to 24 years (*M* = 20.29 years, *SD* = 1.42) with 55% White (*n* = 27), 20% Black/African American (*n* = 10), 16% Asian (*n* = 8), 4% biracial (*n* = 2), and 4% not reported (*n* = 2). We collected data from participants across two sessions, separated by approximately 1 month (*M* = 29.26 days, *SD* = 3.85 days). All 49 participants completed the play-or-pass IGT and self-report measures at session 1 and 39 participants returned for session 2 to complete the IGT and a subset of self-report measures. The study was approved by Temple University’s Institutional Review Board and was performed in accordance with the Declaration of Helsinki.

### Play-or-Pass Iowa Gambling Task

The play-or-pass version of the IGT was administered using E-Prime Stimulus Presentation Software (Schneider et al., 2002). All outcomes in the task were hypothetical. Participants began the task with a “bank” of $2,000. Participants were presented with four decks of cards. On each trial, a single deck was highlighted, and participants had the opportunity to “play” or “pass” on the highlighted deck. If a participant played on a deck, they would receive either a monetary gain, loss, or neither (i.e., $0 change) from the drawn card. If a participant passed on a deck, they moved onto the next trial. Each deck was associated with a different payout distribution such that the average expected value was −$25 for Decks A and B (i.e., the disadvantageous/bad decks) and $20 and $25 for Decks C and D, respectively (i.e., the advantageous/good decks). The order in which participants could play on each deck and the sequence of outcomes associated with each card was fixed across all participants during both sessions; however, participants were not informed of the payout distribution nor the sequence of decks and cards that would be presented. Thus, participants needed to sample cards from each deck to learn the outcomes associated with each deck. In addition, participants were told that their earnings in the task would be exchanged for a real cash bonus.

### Self-report measures

In addition to the IGT, participants completed questionnaires to assess reward/punishment sensitivity and internalizing symptoms. We used the Behavioral Inhibition/Behavioral Activation Scales (BIS/BAS; Carver & White, 1994) to assess reward (behavioral activation) and punishment (behavioral inhibition) sensitivity. The BIS provides a single score of avoidance behavior. The BAS provides a score of general approach behavior as well as scores on subscales to provide specific measures of motivation to pursue goals (BAS Drive), desire and spontaneous approach toward rewards (cf., impulsive behavior; BAS Fun Seeking), and responsiveness to the anticipation and consumption of rewards (BAS Reward Sensitivity). We used the Positive Affect and Negative Affect Schedules (PANAS; Watson et al., 1988), Mood and Anxiety Symptom Questionnaire (MASQ; Watson & Clark, 1991), Snaith-Hamilton Pleasure Scale (SHAPS; Snaith et al., 1995), and the Patient-Reported Outcomes Measurement Information System Depression scale (PROMIS-D) to measure internalizing symptoms (Cella et al., 2007). The PANAS was used to measure positive (PANAS PA) and negative (PANAS NA) affect on the day of data collection. The MASQ was used to measure mood and anxiety in the past week across four subscales: MASQ-General Distress Anxiety which measures anxious mood; MASQ General Distress Depression, which measures depressive mood; MASQ Anxious Arousal, which measures anxiety due to hyperarousal; and MASQ Anhedonic Depression, which measures low positive affect. Finally, the SHAPS was used to measure participants’ ability to experience pleasure in the last few days, and the PROMIS-D was used to measure depression in the past week. The BIS/BAS, PANAS, and SHAPS were administered across both sessions and the MASQ and PROMIS-D were administered only during session 1. Descriptive statistics for these measures are presented Table S1 in the supplemental file.

### Data analysis

We calculated measures of behavior on the play-or-pass IGT using a traditional scoring approach and a computational modeling approach and then tested the reliability and validity of measures from both approaches. First, we evaluated test-retest reliability by calculating the mean-level and rank-order stability of summary scores and computationally-derived task parameters. After evaluating the reliability of the behavioral measures, we examined correlations between behavioral measures of reward and punishment learning. Finally, we evaluated construct validity by estimating correlations between behavioral measures from the IGT with self-reported measures of punishment/reward sensitivity and internalizing symptoms.

Our data and code are available at https://osf.io/ht3sz/?view_only=61b10f605a1749e8989b633203c9a6fa. For traditional scoring, analyses were conducted in R (v. 4.2.2 ; R Core Team, 2022). For computational modeling, we fit the model using hierarchical Bayesian analysis (HBA) in Stan (v. 2.32; Stan Development Team, 2023a, 2023b), a probabilistic programming language, which estimates parameters using Hamiltonian Monte Carlo, a variant of Markov chain Monte Carlo (MCMC), to sample from high-dimensional probabilistic models. We used the RStan package (v. 2.26.22; Stan Development Team, 2023a, 2023b) to interface between R and Stan. We used the wBoot package (Weiss, 2016) to calculate bias-corrected correlation coefficients for assessing reliability of traditional scoring measures and for assessing construct validity (i.e., correlations between IGT & self-report measures; cf., Sullivan-Toole et al., 2022). Bias-corrected correlations were used because some measures were bounded (e.g., learning rates). This approach involves bootstrapping Pearson correlations and estimating a bias-correction parameter from the bootstrap distribution. The correction is then applied to the Pearson correlation coefficient. In addition, we calculated bias-corrected and accelerated confidence intervals which are derived from the same bootstrap distribution of correlations; however, the intervals are adjusted (i.e., accelerated) to account for skewness in the bootstrap distribution. For each correlation, we conducted 9999 replications (the default in wBoot) to generate the bootstrap distribution. For each analysis, we describe mean differences and correlations in which the 95% confidence/credible intervals *do not* overlap with 0 as significantly different or significantly correlated and those that *do* overlap with 0 as not significantly different or not significantly correlated. Given that the behavioral task indices are relatively novel and that no power analysis was conducted to determine a necessary sample size, we recommend that readers reference the relevant visual presentations of data, in addition to the statistical analyses, to judge how meaningful a given result is, rather than relying solely on binary conclusions of statistical significance.

### Traditional scoring approach

Traditional scoring involved calculating the proportion of plays on good (i.e., advantageous) decks and bad (i.e., disadvantageous) decks, separately. Session-wide play proportions on good and bad decks represent gross measures of reward and punishment learning, respectively. Specifically, more plays on the good decks reflect higher reward learning (i.e., approach of reward) and fewer plays on the bad decks reflect higher punishment learning (i.e., avoidance of punishment). To evaluate mean-level stability, we used paired-samples *t*-tests to compare the proportion of plays between sessions 1 and 2, separately for good and bad decks. To evaluate rank-order stability, we calculated correlations between the proportion of plays during sessions 1 and 2, separately for good and bad decks. Next, we correlated the proportion of plays on good decks with the proportion of plays on bad decks to determine whether these measures could dissociate reward and punishment learning. Finally, we evaluated construct validity by correlating the proportion of plays on good and bad decks with scores on each of the self-report measures.

### Computational modeling approach

For the computational model, we fit a modified version of the Outcome-Representation Learning (ORL) model. The ORL is a generative model that was developed to analyze choice in the original IGT in which all four decks are presented simultaneously and participants play on one of the four decks on each trial (Haines et al., 2018; Sullivan-Toole et al., 2022). In the present study, participants were presented with decks one at a time and would either play or pass on the presented deck within each trial. Thus, we modified the original ORL to accommodate the play-or-pass nature of this version of the IGT. Specifically, choices to play or pass were modeled as a function of the value of playing on that deck using a logistic function:

1 $${Y}_{j}(t)\sim bernoulli\left(\frac{1}{1+exp(-{V}_{j}\left(t\right))}\right)$$

where ${Y}_{j}\left(t\right)$ indicates whether the participant played (${Y}_{j}\left(t\right)=1$) versus passed (${Y}_{j}\left(t\right)=0$) when presented with deck *j* on trial *t*, and *V*_*j*_(*t*) is the value of playing when presented with deck *j* on trial *t*. This choice rule implies that the value of passing is always held constant at 0—only the value of playing is updated on a trial-by-trial basis. Specifically, after each choice, *V*_*j*_ is updated according to the following equation:

2 $${V}_{j}\left(t+1\right)= {EV}_{j}\left(t+1\right)+{EF}_{j}\left(t+1\right)\bullet \beta f+\beta b$$

where *V*_*j*_(*t* + 1) is the value of playing on deck *j* on the next trial (i.e., *t* + 1), *EV*_*j*_(*t* + 1) is the expected outcome value associated with playing or passing on deck *j* in the next trial, *EF*_*j*_(*t* + 1) is the expected win frequency of playing or passing on deck *j* in the next trial, *βf* is a free parameter describing sensitivity to win frequency, and *βb* is a free parameter describing bias towards playing (when positive) or passing (when negative), regardless of the deck.

The expected outcome value (*EV*_*j*_) and expected win frequency (*EF*_*j*_) are updated from trial to trial based on the outcome received after playing on deck *j*. Specifically, the expected outcome value for the next trial (i.e., *t* + 1) is calculated by

3 $${EV}_{j}\left(t+1\right)=\left\{\begin{array}{l}{EV}_{j}\left(t\right)+{A}_{rew}\bullet \left(x\left(t\right)-{EV}_{j}\left(t\right)\right)\ \ \ \ \ \ \ if\ {Y}_{j}\left(t\right)=1 \text{and}\ x\left(t\right)\ge 0\\ {EV}_{j}\left(t\right)+{A}_{pun}\bullet \left(x\left(t\right)-{EV}_{j}\left(t\right)\right) \ \ \ \ \ \ \ if\ {Y}_{j}\left(t\right)=1\ \text{and}\ x\left(t\right)<0\\ {EV}_{j}\left(t\right)\ \ \ \ \ \ \ \ \ \ \ \ \ \ \ \ \ \ \ \ \ \ \ \ \ \ \ \ \ \ \ \ \ \ \ \ \ \ \ \ \ \ \ \ \ \ \ \ \ \ \ \ if\ {Y}_{j}\left(t\right)=0\end{array}\right.$$

where *EV*_*j*_(*t*) is the expected outcome value of playing on deck *j* in the current trial (i.e., *t*), *x*(*t*) is the amount of the gain or loss, *A*_*rew*_ is a free parameter describing learning rate for gains (i.e., when *x*(*t*) > 0), and *A*_*pun*_ is a free parameter describing learning rate for losses (i.e., when *x*(*t*) < 0). The expected win frequency for the next trial is calculated by

4 $${EF}_{j}\left(t+1\right)=\left\{\begin{array}{l}{EF}_{j}\left(t\right)+{A}_{rew}\bullet \left(sgn\left(x\left(t\right)\right)-{EF}_{j}\left(t\right)\right)\ \ \ \ \ \ \ \ \ \ \ \ \ \ \ if\ {Y}_{j}\left(t\right)=1\ \text{and}\ x\left(t\right)\ge 0\\ {EF}_{j}\left(t\right)+{A}_{pun}\bullet \left(sgn\left(x\left(t\right)\right)-{EF}_{j}\left(t\right)\right)\ \ \ \ \ \ \ \ \ \ \ \ \ \ if\ \ {Y}_{j}\left(t\right)=1\ \text{and}\ x\left(t\right)<0\\ {EF}_{j}\left(t\right)\ \ \ \ \ \ \ \ \ \ \ \ \ \ \ \ \ \ \ \ \ \ \ \ \ \ \ \ \ \ \ \ \ \ \ \ \ \ \ \ \ \ \ \ \ \ \ \ \ \ \ \ \ \ \ \ \ \ \ \ \ \ \ \ \ \ \ \ \ if\ {Y}_{j}\left(t\right)=0\end{array}\right.$$

where *EF*_*j*_(*t*) is the expected win frequency of playing on deck *j* in the current trial, *A*_*rew*_ and *A*_*pun*_ are as described above, and the *sgn*(*x*(*t*)) term refers to the sign of the outcome (using the signum function).^2^ In addition to updating the expected win frequency of the current deck, the expected win frequencies for playing on the other decks are updated according to the following fictive updating rule:

5 $${EF}_{'j}\left(t+1\right)\left\{\begin{array}{l}{EF}_{'j}\left(t\right)+A_{pun}\bullet\left(\frac{-sgn\left(x\left(t\right)\right)}C-EF_{j}^{^{\prime}}\left(t\right)\right)\;\;\;\;\;\;\;if\;Y_j\left(t\right)=1\;\text{and}\;x\left(t\right)\geq0\\{EF}_{'j}\left(t\right)+A_{rew}\bullet\left(\frac{-sgn\left(x\left(t\right)\right)}C-EF_j^{^{\prime}}\left(t\right)\right)\;\;\;\;\;\;\;if\ Y_j\left(t\right)=1\ \text{and}\ x\left(t\right)<0\\{EF}_{'j}\left(t\right)\;\;\;\;\;\;\;\;\;\;\;\;\;\;\;\;\;\;\;\;\;\;\;\;\;\;\;\;\;\;\;\;\;\;\;\;\;\;\;\;\;\;\;\;\;\;\;\;\;\;\;\;\;\;\;\;if\ Y_j\left(t\right)=0\end{array}\right.$$

where *EF*_*’j*_(*t*) is the expected win frequency of the other decks during the current trial, *C* is the number of other decks available (i.e., 3), and all other terms are as described above. The parameterization of this version of the ORL model is the same as the original model, except we removed memory decay (*K*) from the original model, and we replaced *βp* (perseverance) from the original model with *βb*, bias. We chose this parameterization because when we fit the original ORL model to this data, *βp* functioned similar to bias (i.e., was associated with generally playing/passing more frequently), and memory decay showed poor reliability as well as poor recovery when we conducted parameter recovery diagnostics. Details of fitting the original ORL model and the parameter recovery diagnostics are in the supplemental file. In summary, the ORL model for the play-or-pass IGT has four free parameters, *A*_rew_, *A*_*pun*_, *βf*, and *βb*, that allow us to capture individual-differences related to task performance.

As described above, we used a joint modeling approach to model choice data from both sessions simultaneously in a single model.^3^ We estimated *A*_rew_, *A*_*pun*_, *βf*, and *βb* hierarchically such that parameters were allowed to vary for each participant within each session (i.e., random effects for participants). Fitting the model hierarchically in this fashion allowed for information across individuals and sessions to be pooled across person-level parameters which causes person-level estimates to regress toward the group-level mean. For each free parameter (e.g., *βf*), person-level parameter estimates were assumed to follow a multivariate normal distribution, given by the following:

6 $$\left[\begin{array}{c}{\theta }_{i,1}\\ {\theta }_{i,2}\end{array}\right]\sim MVNormal\left(\left[\begin{array}{c}{\mu }_{\theta ,1}\\ {\mu }_{\theta ,2}\end{array}\right] , {\mathbf{S}}_{\theta }\right)$$

where *θ*_*i1*_ and *θ*_*i2*_ are the person-level parameters for participant *i* on sessions 1 and 2, respectively; *θ* refers to either `*A*_rew_, `*A*_*pun*_, *βf*, or *βb*; *μ*_*θ1*_ and *μ*_*θ2*_ are the group-level parameter means for sessions 1 and 2, respectively; and **S**_***θ***_ is the covariance matrix for session 1 and 2 person-level parameters. In the ORL, *A*_*rew*_ and *A*_*pun*_ are bounded between 0 and 1; thus, `*A*_rew_ and `*A*_*pun*_ were estimated assuming a multivariate normal distribution (Eq. 6) and then transformed using the inverse cumulative normal distribution to obtain *A*_*rew*_ and *A*_*pun*_ (Haines et al., 2018). The covariance matrix, **S**_***θ***_, can be decomposed into a 2 × 2 matrix of the group-level standard deviations for each session (*σ*_*θ*,1_ & *σ*_*θ*,2_) and a 2 × 2 correlation matrix (**R**_***θ***_):

7 $${\mathbf{S}}_{{\varvec{\theta}}}=\left[\begin{array}{cc}{\sigma }_{\theta ,1}& 0\\ 0& {\sigma }_{\theta ,2}\end{array}\right]{\mathbf{R}}_{{\varvec{\theta}}}\left[\begin{array}{cc}{\sigma }_{\theta ,1}& 0\\ 0& {\sigma }_{\theta ,2}\end{array}\right]$$

where

8 $${\mathbf{R}}_{{\varvec{\theta}}}=\left[\begin{array}{cc}{\rho }_{\theta 12}& 0\\ 0& {\rho }_{\theta 12}\end{array}\right]$$

and *ρ*_*θ12*_ is the correlation between *θ* on session 1 and *θ* on session 2 (i.e., the test-retest reliability estimate).

For each parameter, we used weakly informative priors. Priors for group-level means on sessions 1 and 2 in Eq. 6 were specified as

**Table:** 

μ_θ1_	~ Normal(0,1)
μ_θ2_	~ Normal(0,1)

for `*A*_rew_, `*A*_*pun*_, *βf*, and *βb*. Priors for group-level standard deviations on sessions 1 and 2 in Eq. 7 were specified as

**Table:** 

σ_θ1_	~ Half-Normal(0.2)
σ_θ2_	~ Half-Normal(0.2)

for `*A*_rew_ and `*A*_*pun*_ and as

**Table:** 

σ_θ1_	~ Half-Cauchy(1)
σ_θ2_	~ Half-Cauchy(1)

for *βf* and *βb*. We chose stronger, half-normal priors on the *σ*s for `*A*_rew_ and `*A*_*pun*_ to avoid extreme values of these parameters after transforming. The priors for the correlation matrices were specified as

**Table:** 

R_θ12_	~ LKJcorr(1)

for each parameter. Finally, the session 1 and session 2 group-level means and standard deviations served as the priors for the person-level parameters on sessions 1 and 2, respectively, such that

**Table:** 

θ_i1_	~ Normal(μ_θ1_, σ_θ1_)
θ_i2_	~ Normal(μ_θ2_, σ_θ2_)

for *βf* and *βb*, and as

**Table:** 

Φ^–1^(*θ*_*i1*_ / *scale*)	~ Normal(*μ*_*θ1*_,* σ*_*θ1*_)
Φ^–1^(*θ*_*i2*_ / *scale*)	~ Normal(*μ*_*θ2*_,* σ*_*θ2*_)

for *A*_rew_ and *A*_*pun*_. Here, Φ^–1^ is the inverse of the cumulative distribution function of the normal distribution, and *scale* indicates a scaling factor applied to the parameter to ensure it meets the appropriate parameter bound. For *A*_rew_ and *A*_*pun*_ , *scale* = 1, resulting in the learning rates being bounded between 0 and 1.

For clarity, we write the centered parameterizations above; however, we implemented the model by using noncentered parameterizations to improve convergence and estimation efficiency. Similarly, to improve sampling from the multivariate normal distributions, we used Cholesky decompositions of the correlation matrices (Haines et al., 2018). Details of the noncentered parameterizations are in the supplement. We sampled the model by using 4 chains, each with 5,000 iterations with the first 1,000 iterations as warmup (Sullivan-Toole et al., 2022). After fitting the model, we checked for convergence of target distributions visually with trace-plots and for each parameter numerically with $\widehat{R}$ values (Gelman & Rubin, 1992). $\widehat{R}$ values were all <1.1, indicating that the variance between chains did not outweigh the variance within chains (i.e., convergence). We performed posterior predictive checks by simulating data from the model and visually inspected how well the model fit the data by comparing simulated data with the observed data. Finally, we performed parameter recovery diagnostics which showed adequate recovery of all parameters. Parameter recovery diagnostics are displayed in the supplement.

Because we used a Bayesian approach, we based our inferences off evaluating the posterior distributions of parameters. We assessed mean-level stability by calculating differences between session 1 and session 2 group-level posterior distributions for each parameter. We assessed rank-order stability by examining the posterior distributions of the *ρ*_*θ12*_-values from Eq. 8 for each parameter. We computed correlations between the person-level posterior means of reward and punishment learning rates to determine whether these measures could dissociate reward approach and punishment learning. Finally, to evaluate construct validity, we correlated the posterior means of each person-level parameter with scores on each of the self-report measures. As described above, we calculated bootstrapped correlations with bias-corrected and accelerated bootstrapped confidence intervals for each correlation except for the assessment of rank-order stability which was estimated within the joint model.

## Results

### Test-retest reliability

#### Traditional scoring

**Fig. 1:**
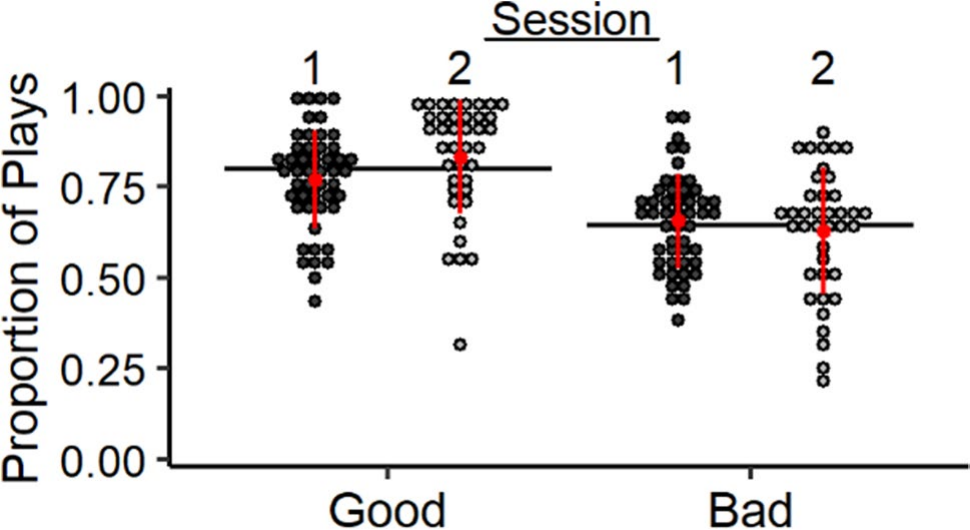
Mean-level stability of traditional scoring. *Note.* Proportion of plays on good and bad decks. Horizontal bars represent group means collapsed across sessions. Red datapoints and error bars represent session-specific means ±1 SD. Finally, individual datapoints represent person-level play proportions during session 1 (filled datapoints) and session 2 (open datapoints)

Overall, participants were sensitive to the higher payoff structure of the good decks relative to the bad decks. To illustrate, Fig. 1 shows that the proportion of plays on the good decks was higher than for the bad decks. Table 1 shows session-specific descriptive statistics and estimates of test-retest reliability for the IGT measures. We first examined mean-level between-session stability of the proportion of plays on good decks and found a small, but significant increase in plays on the good decks between session 1 and 2, *t*(38) = 3.24, CI [.02, .10]. The proportion of plays on bad decks decreased between sessions; however, this difference was not significant, *t*(38) = −.93, CI [−.07, .03]. Thus, participants increased playing on good decks more frequently across sessions, but participants did not significantly change behavior on bad decks across sessions.

**Table 1:** Descriptive statistics and test-retest reliability estimates for IGT measures

	Proportion of plays		ORL parameters			
Estimate	Good decks	Bad decks	*A+*	*A−*	*βf*	*βb*
Session 1 *M* [95% CI]	.77 [.73, .81]	.66 [.62, .69]	.17 [.13, .21]	.10 [.08, .12]	2.42 [1.44, 3.36]	0.77 [0.56, 0.99]
Session 2 *M* [95% CI]	.83 [.78, .88]	.63 [.57, .68]	.10 [.07, .13]	.10 [.08, .12]	2.75 [1.28, 4.21]	1.44 [1.11, 1.76]
Session 2–1 *M* [95% CI]	**.06 [.02, .10]**	−.02 [−.07, .03]	**−.07 [−.12, −.03]**	.00 [-.03, .02]	0.32 [-1.06, 1.87]	**0.67 [0.34, 1.00]**
*r* [95% CI]	**.69 [.45, .81]**	**.49 [.22, .67]**	**.67 [.07, 1.00]**	**.66 [.27, 1.00]**	**.59 [.28, .86]**	**.60 [.24, .90]**

Confidence/credible intervals for mean differences and correlations that do not overlap with 0 are bolded

**Fig. 2:**
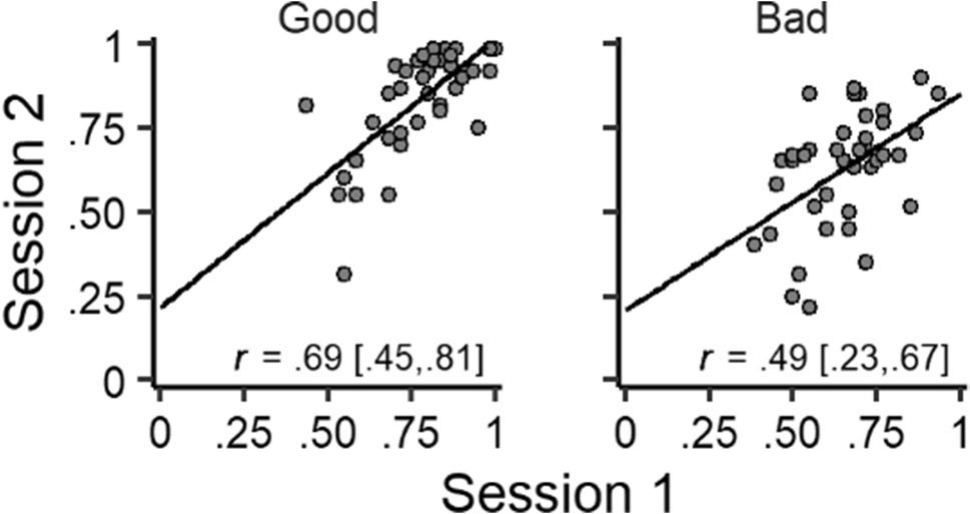
Rank-order stability of traditional scoring. *Note.* Proportion of plays on good (left) and bad (right) decks during session 2 as a function of the proportion of plays on those decks during session 1

Next, we examined the rank-order stability of playing on the good and bad decks. Figure 2 shows the proportion of plays on the good (left) and bad (right) decks during session 2 as a function of the proportion of plays on those decks during session 1. For both good and bad decks, the proportion of plays during session 1 was positively related to the proportion of plays during session 2 (Table 1). Thus, participants who played on either a good or bad deck more frequently during session 1 also played on that deck more frequently during session 2.

#### Computational model parameters

**Fig. 3:**
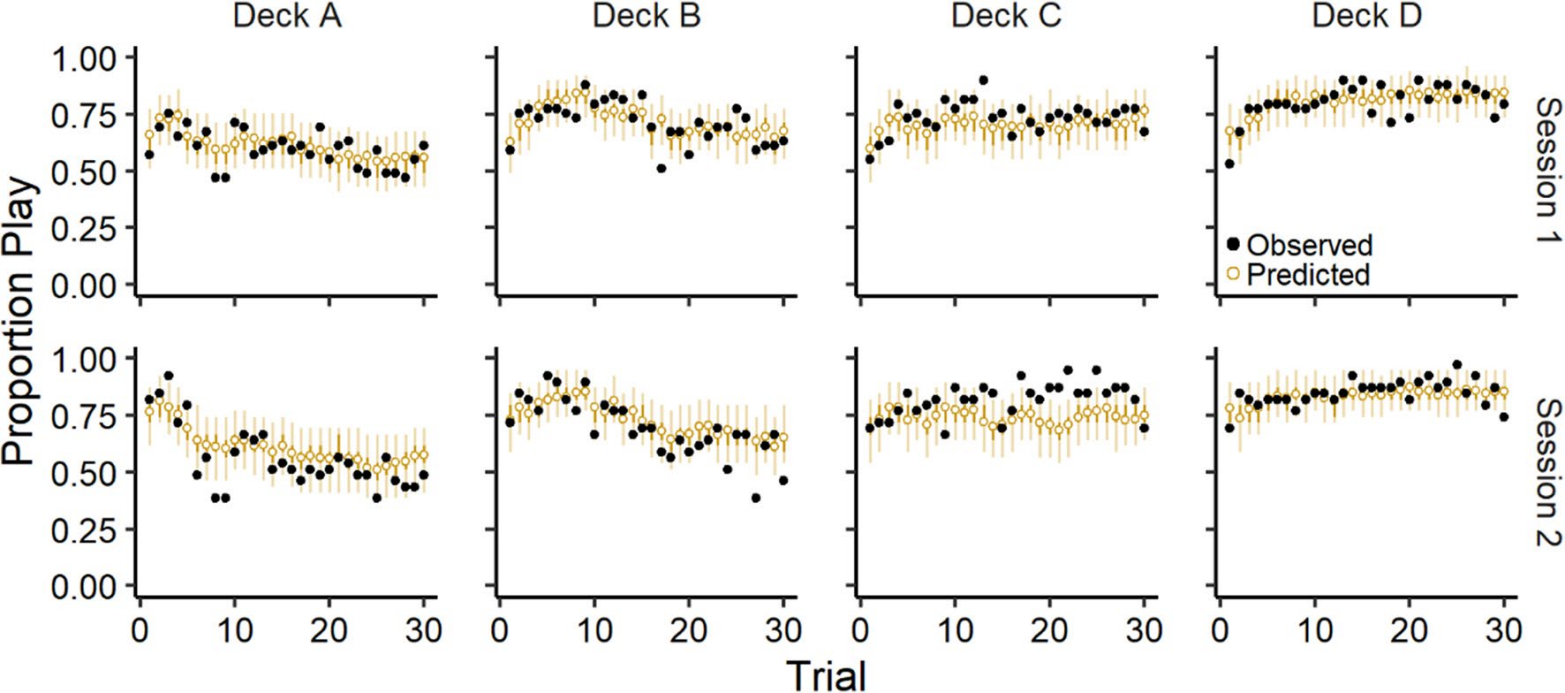
Group-level proportion of plays across trials for each deck. *Note.* Group-level mean observed and ORL model-predicted play proportions across trials for each deck (columns) during each session (rows). Error bars represent 50% (darker) and 95% (lighter) credible intervals

In Fig. 3, we present the observed and ORL model-predicted proportion of plays on each trial for the good decks (C & D) and bad decks (A & B), averaged across participants for each session. Overall, participants tended to play more frequently on the good decks and less frequently on the bad decks across trials and predicted values from the ORL model were consistent with these overall trends.

**Fig. 4:**
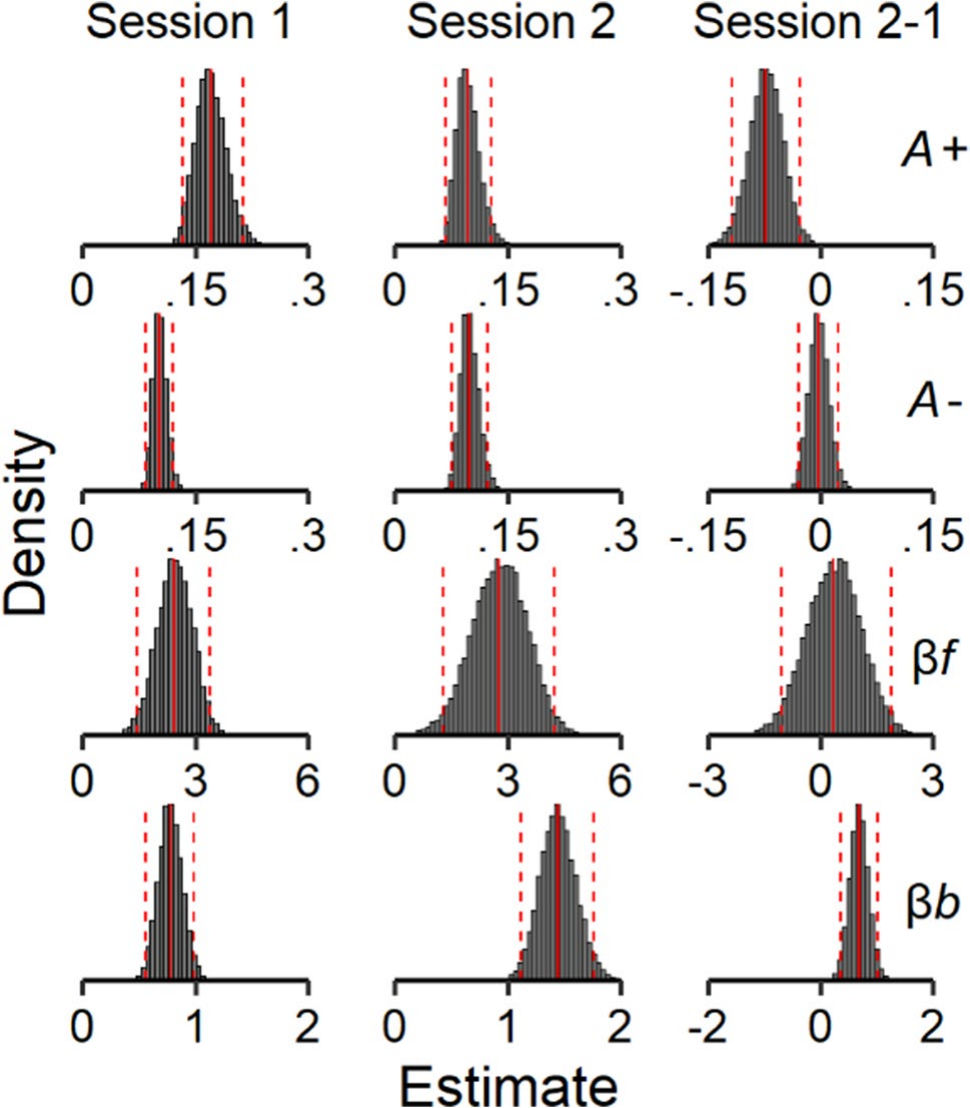
Mean-level stability of ORL parameters. *Note.* Posterior distributions of the group-level ORL parameters from session 1 (left) and session 2 (middle), as well as the difference between session 1 and session 2 estimates (right). Solid red vertical lines represent the posterior means, and the dashed red vertical lines represent the lower and upper bounds of the 95% credible intervals

**Fig. 5:**
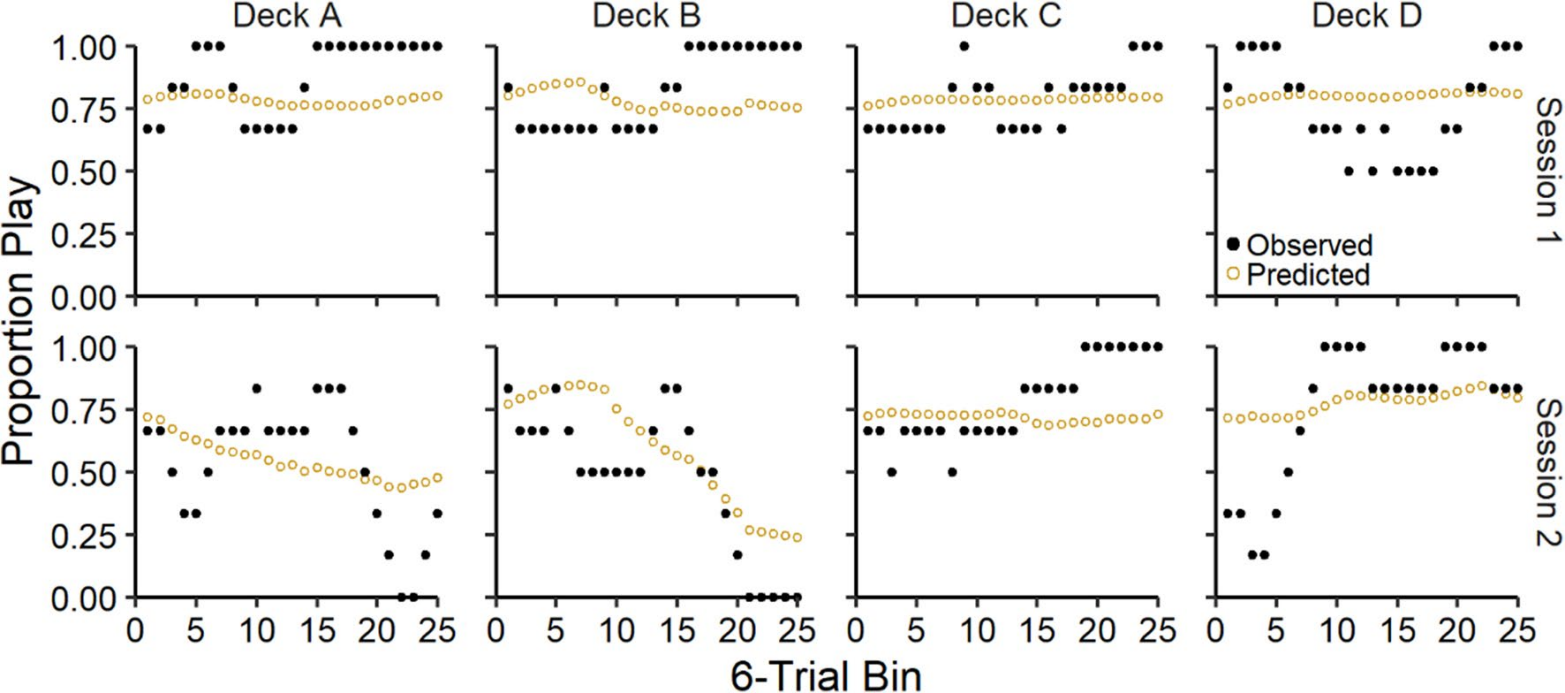
Proportion of plays across trials for each deck for participant 2051*. Note.* Person-level observed and predicted play proportions across trials for each deck (columns) for participant 2051 across both sessions (rows). Observed and predicted proportions represent smoothed averages with a 6-trial window

**Fig. 6:**
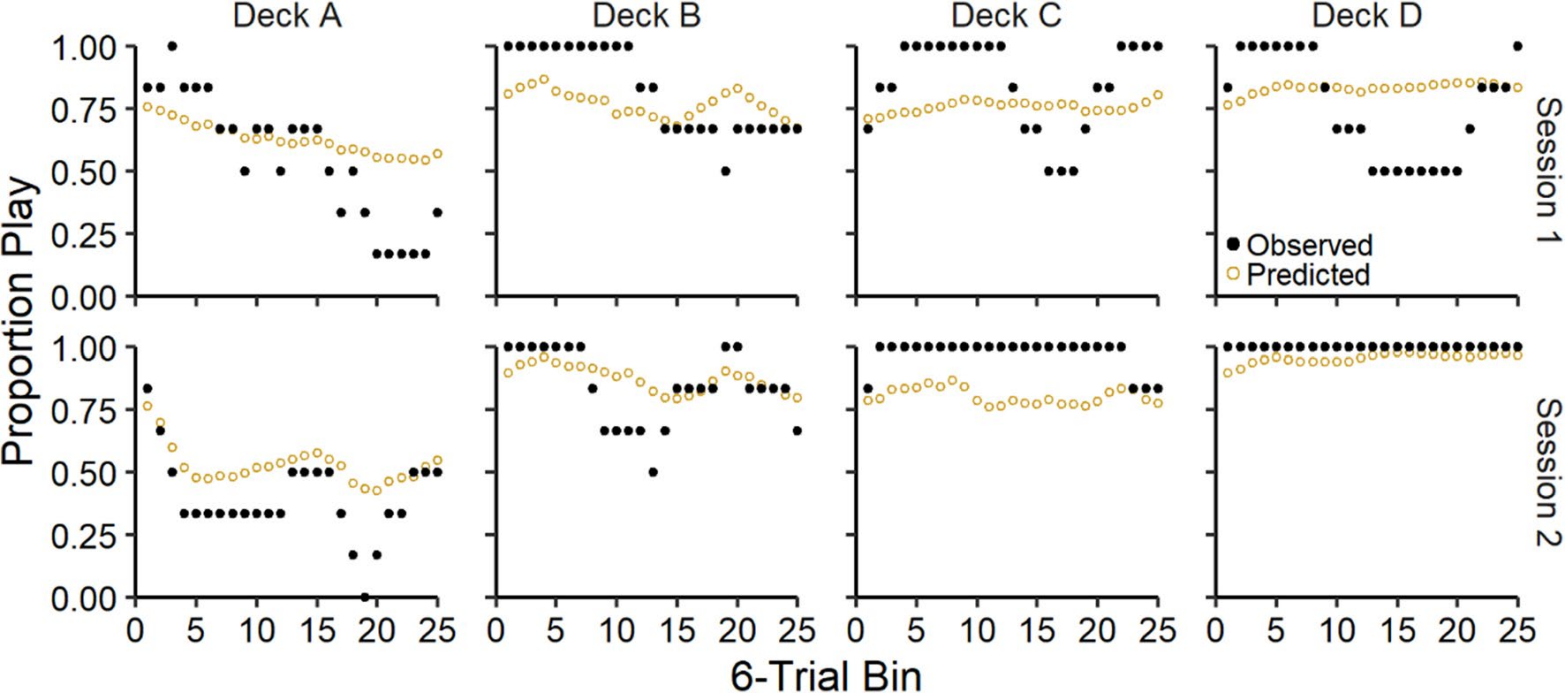
Proportion of plays across trials for each deck for participant 2050. *Note.* Person-level observed and predicted play proportions across trials for each deck (columns) for participant 2050 across both sessions (rows). Observed and predicted proportions represent smoothed averages with a 6-trial window

First, we examined the mean-level stability of the ORL parameters. Figure 4 shows the posterior distributions of each group-level mean parameter for sessions 1 and 2, as well as the posterior distributions of the difference between sessions 1 and 2 for each group-level mean parameter (Table 1). Punishment learning rate (*A-*) and win frequency sensitivity (*βf*) were similar across sessions; the 95% credible intervals for the between-session differences in these parameters overlapped with 0 (see right column in Fig. 4). In contrast, reward learning rate (*A+*) decreased, and bias (*βb*) increased from session 1 to session 2; the 95% credible intervals for the between-session differences in these parameters *did not* overlap with 0. To illustrate the behavioral changes associated with the decreases in reward learning rate and increases in bias across sessions, we present data from two participants that are representative of these changes. Figure 5 shows a participant that illustrates the behavioral changes associated with decreases in reward learning rate. Specifically, decreases in reward learning rates were generally associated playing less frequently on bad decks *across trials*; this participant played on bad decks throughout session 1 but showed a decreasing pattern of playing on the bad decks across trials during session 2. Figure 6 shows a different participant that illustrates the behavioral changes associated with increases in bias. Specifically, increases in the bias parameters were associated with increases in plays across all decks; this participant played more frequently in general, except for Deck A, during the second session relative to the first. These two participants are representative of the behavioral changes associated with the changes in these ORL parameters; however, we also present results from a more detailed simulation of these effects, as well as data from all participants, in the supplemental file.

**Fig. 7:**
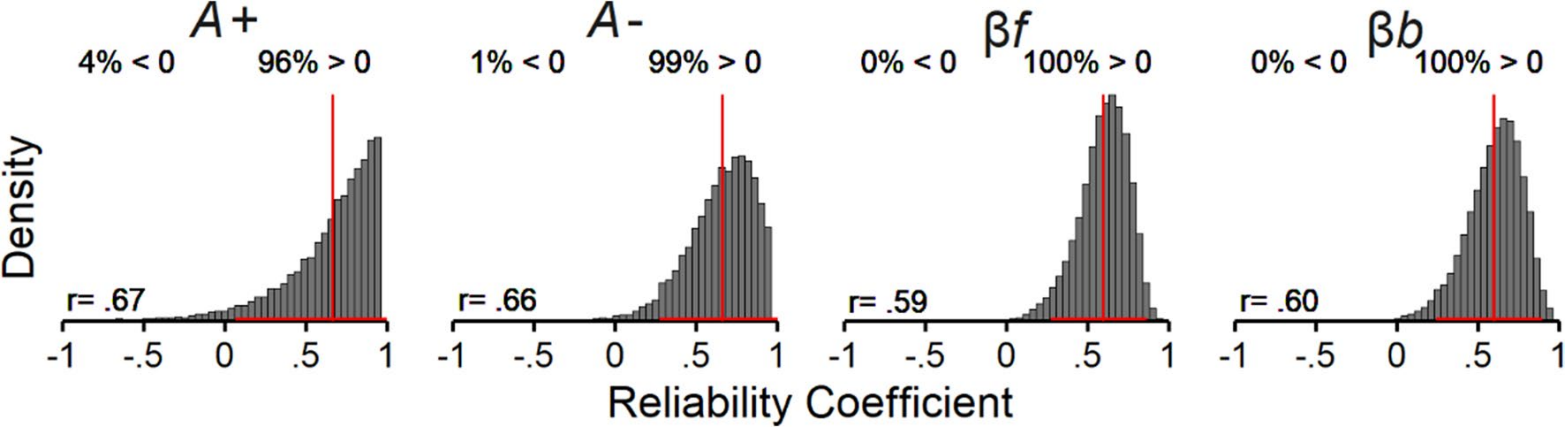
Rank-order stability of ORL parameters. *Note.* Posterior distributions of the reliability coefficients estimated for each parameter in the joint ORL model. Solid red vertical lines represent the posterior means (presented at the bottom left of each panel), horizontal lines represent the lower and upper bounds of the 95% credible intervals, and values to the left and rights sides of each panel represent the % of reliability estimates below and above 0, respectively

Next, we examined the rank-order stability of the ORL model parameters. Figure 7 shows the posterior distributions of the reliability coefficients for each parameter (Table 1). The posterior means of the reliability coefficients for each parameter were moderate to strong (Cohen, 1988, 1992), and the 95% credible intervals did not overlap with 0. Thus, the ORL model parameters showed good rank-order stability.

#### Dissociation of reward and punishment learning

Measures of reward and punishment learning were unrelated using both approaches. Specifically, although moderate in magnitude, the proportion of plays on good and bad decks (traditional scoring approach) were not significantly correlated within either session, session 1: *r* = .27, CI [−.06, .55], session 2: *r* = .33, CI [−.01, .68]. The reward and punishment learning rates (computational modeling approach) also were not correlated within either session, session 1: *r* = .07, CI [−.17, .40], session 2: *r* = .04, CI [−.23, .33]. Thus, both approaches capture the dissociation between reward and punishment learning.

#### Correlations with self-report measures

To examine the relation between IGT behavioral measures and self-reported internalizing symptoms, we collapsed (i.e., averaged) behavioral measures and self-report scores across sessions. We collapsed measures across sessions because we were primarily interested in correlations between stable trait measures. Correlations at the session-specific level are presented in the supplemental file. For the traditional scoring approach, the proportion of plays on bad decks were negatively correlated with scores on the PANAS NA (Table 2). For the computational modeling approach, we found that reward learning rates (*A+*) were negatively correlated with scores on the PANAS NA, punishment learning rates (*A-*) were positively correlated with scores on the BAS fun seeking subscale, and estimates of win frequency sensitivity (*βf*) were negatively correlated with scores on the BAS drive subscale. There were no significant associations between self-report measures and the proportion of plays on good decks and estimates of bias. Thus, measures of punishment learning across both the traditional and computational modeling approaches were related to the self-report measures; however, only the computationally-derived measure of reward learning showed associations with self-report measures.

**Table 2:** Correlations between Self-Report Scores and IGT Measures

	Proportion of plays		ORL Parameters			
Self-Report	Good decks	Bad decks	*A+*	*A−*	*βf*	*βb*
BAS total	−.14 [−.42, .22]	-.00 [−.29, .30]	.19 [−.19, .45]	.07 [−.21, .33]	−.13 [−.37, .13]	−.07 [−.34, .22]
BAS drive	−.23 [−.54, .27]	.09 [−.22, .43]	.25 [−.11, .56]	−.06 [−.47, .23]	**−.26 [−.50, −.02]**	−.17 [−.44, .23]
BAS fun seeking	.08 [−.26, .42]	−.14 [−.50, .25]	−.01 [−.38, .31]	**.27 [.03, .46]**	−.02 [−.25, .23]	.11 [−.27, .43]
BAS reward responsivity	−.16 [−.38, .08]	.02 [−.21, .27]	.18 [−.06, .40]	−.01 [−.27, .28]	.01 [−.26, .31]	−.07 [−.28, .15]
BIS total	.11 [−.27, .40]	−.10 [−.38, .21]	−.27 [−.53, .02]	.03 [−.24, .24]	.14 [−.11, .39]	.16 [−.17, .43]
PANAS PA	−.01 [−.26, .25]	.13 [−.22, .38]	.17 [-.10,.40]	.08 [−.17, .35]	−.17 [-.40,.09]	.06 [−.23, .35]
PANAS NA	−.19 [−.40, .06]	**−.29 [−.53, −.01]**	**−.21 [−.38, −.01]**	.23 [−.05, .54]	−.18 [−.42, .06]	−.11 [−.34, .12]
MASQ general distress anxiety	.03 [−.19, .27]	−.15 [−.42, .14]	−.19 [−.41, .06]	.20 [−.12, .55]	−.01 [−.28, .26]	.10 [−.13, .32]
MASQ general distress depression	.04 [−.23, .29]	−.13 [−.41, .27]	−.06 [−.28, .15]	.16 [−.20, .55]	−.16 [−.43, .11]	.01 [−.19, .22]
MASQ anxious arousal	-.01 [−.24, .24]	−.12 [−.42, .15]	−.10 [−.29, .09]	.23 [−.11, .53]	.02 [-.22, .22]	.07 [−.19, .35]
MASQ Anhedonic depression	−.01 [−.35, .30]	−.08 [−.36, .30]	−.25 [−.49, .01]	−.16 [−.44, .11]	.05 [−.19, .30]	−.04 [−.38, .31]
SHAPS	−.10 [−.34, .15]	−.02 [−.35, .23]	−.00 [−.49, .30]	.11 [−.07, .30]	−.17 [−.47, .25]	−.08 [−.36, .15]
PROMIS-D	−.03 [−.39, .30]	−.10 [−.39, .28]	−.20 [−.44, .04]	−.10 [−.36, .14]	.02 [−.23, .26]	−.01 [−.35, .33]

Correlations with bootstrapped 95% confidence intervals. Confidence intervals that do not overlap with 0 are bolded

## Discussion

This study examined the psychometric properties of the play-or-pass version of the Iowa Gambling Task (IGT) using a traditional scoring and a computational modeling approach. For the traditional scoring approach, we calculated the proportion of plays on good and bad decks. For the computational modeling approach, we fit a modified version of the Outcome Representation Learning (ORL) model to derive four measures of task behavior: reward (*A+*) and punishment (*A−*) learning rates, win frequency sensitivity (*βf*), and bias (*βb*). Using both approaches, we characterized test-retest reliability in terms of the mean-level and rank-order stability of behavior across two sessions. We found that most but not all measures were stable across the two sessions. Next, we examined whether these approaches could dissociate reward and punishment learning by examining correlations between the proportion of plays on good and bad decks (traditional approach) and between reward and punishment learning rates (computational approach). For both approaches, reward and punishment learning was dissociable. Finally, we examined whether measures from the traditional scoring and computational modeling approaches were related to internalizing symptoms. Measures from both approaches were related to internalizing symptoms; however, we found that measures from the computational modeling approach were associated with a wider range of symptoms. We discuss each of these results in further detail below.

We evaluated test-retest reliability by first examining mean-level stability of measures from the traditional and computational modeling approaches. For the traditional scoring approach, the proportion of plays on good decks significantly increased across sessions, and the proportion of plays on bad decks decreased across sessions although to a small and nonsignificant degree. These findings indicate that participants made more “optimal” choices (i.e., chose decks with higher long-term payoffs more frequently) after gaining experience with the task. For the computational modeling approach, the average estimate of reward learning rate decreased, and bias increased across sessions. Increases in bias capture increases in playing on all decks, and decreases in reward learning rate were associated with decreases in playing on bad decks specifically. Combined, this pattern of changes in the ORL parameters relates to an increase in plays on good decks but only a small change in plays on bad decks across sessions. Sullivan-Toole et al. (2022), who used the ORL model to characterize test-retest reliability for the traditional IGT, found no between-session changes in either ORL parameters or choice proportions. This could indicate that the play-or-pass version of the IGT may be better equipped to capture between-session changes in decision-making; however, others have found more optimal decision-making across time using the traditional IGT (Bechara et al., 1994; Buelow & Barnhart, 2018). Such changes could be important for detecting deficits in decision-making. For example, Bechara et al. noted that participants *without* prefrontal cortex damage showed improved performance on the traditional IGT across time but that individuals *with* prefrontal cortex damage did not show changes in performance across time. Thus, if the play-or-pass IGT can capture changes in optimal decision making across sessions better than the traditional IGT, this could have implications for using the play-or-pass IGT as an assessment tool.

Next, we examined the rank-order stability of measures from the traditional and computational modeling approaches. For the traditional scoring approach, the proportion of plays on good and bad decks were significantly correlated between sessions 1 and 2 with moderate to strong positive correlations (Cohen, 1988, 1992). Some studies find relatively low reliability for similar measures from the traditional IGT (e.g., *r* ~ .30; Buelow & Barnhart, 2018; Schmitz et al., 2020); thus, our results are promising in that the play-or-pass IGT produces more reliable measures of decision-making at a session-wide level. For the computational modeling approach, estimates of reliability for reward and punishment learning rates, win frequency sensitivity, and bias were also moderate to strong. These findings are consistent with Sullivan-Toole et al. (2022) who used the ORL model in a joint modeling framework for the traditional IGT and found strong estimates of reliability for ORL model parameters. Comparing across these studies should be made with caution as we modified the ORL model from its original parameterization. Specifically, we removed the memory decay and perseverance parameters from the original ORL model and added a bias parameter, similar to a go-bias parameter used in Pavlovian go/no-go tasks (Cavanagh et al., 2013). Because this study is a preliminary extension of the ORL model to the play-or-pass IGT, future research should test whether the modified ORL model (with bias) or the original ORL model (with memory decay & perseverance) best characterize data from the play-or-pass IGT, particularly in other samples (e.g., children).

We also examined associations between reward and punishment learning with the traditional and computational modeling approaches. Across both approaches, correlations were non-significant, indicating that reward and punishment learning were dissociable on the play-or-pass IGT. When considering the magnitude of the associations between reward and punishment learning, however, correlations using the traditional scoring approach were moderate whereas correlations using the computational modeling approach were small. A purported advantage of the play-or-pass IGT is that it allows us to better separate reward and punishment learning using traditional scoring procedures. Overall, this was supported; however, we also show that the ORL model may more effectively separate these forms of learning. It should be noted that these approaches for characterizing learning are qualitatively different. For the traditional scoring approach, the proportion of plays on good and bad decks reflects an individual’s behavior toward specific decks as a whole (i.e., whether they learned the deck to be advantageous [i.e., “good”] or disadvantageous [i.e., “bad”] on average). For the computational modeling approach, reward and punishment learning rates reflect an individual’s behavior across all decks (i.e., how influential a rewarding or punishing outcome is on the individual’s subsequent choices regardless of whether the deck is advantageous or disadvantageous). Depending on the specific research question, a researcher may be interested in one or both aspects of how these analytical techniques characterize reward and punishment learning.

Finally, we examined correlations between measures from both analytical approaches with self-reported measures of reward/punishment sensitivity and internalizing symptoms. With the traditional scoring approach, we found that the proportion of plays on bad decks was negatively correlated with self-reported state-level negative affect (PANAS NA scores). This is opposite of Sullivan-Toole et al.’s (2022) findings in which state-level negative affect was associated with more frequent choices of disadvantageous decks on the original IGT. This could indicate that summary measures from the original and play-or-pass IGT capture different aspects of affective decision-making. For example, individuals with higher negative affect may be more sensitive to negative feedback on the play-or-pass IGT, and thus more readily avoid the bad decks (Haines et al., 2021). In contrast, individuals with higher negative affect may engage in more risky decision-making on the original IGT, and thus choose the bad decks more frequently (Leith & Baumeister, 1996). Future research could explore these between-task differences by examining how affect manipulations (e.g., mood induction) influence decision-making on the original and play-or-pass IGT.

With the computational modeling approach, we found several significant correlations between the self-report measures and parameters from the ORL model. First, we found that punishment learning rates were positively associated with self-reported fun-seeking on the BAS. That is, individuals who reported engaging in more fun-seeking behaviors were also more sensitive to negative outcomes in the IGT. Similar comparisons show mixed findings in the literature regarding the relation between punishment sensitivity and fun-seeking (Case & Olino, 2020; Loxton et al., 2008); thus, further research is necessary to clarify this relation. Next, we found that reward learning rates were negatively correlated with state-level negative affect (PANAS NA scores), which is consistent with the traditional scoring approach because, as described above, lower reward learning rates were associated with sharper decreases in playing on the bad decks across trials. Finally, estimates of win frequency sensitivity were negatively correlated with BAS Drive scores. Win frequency sensitivity is associated with playing on decks with more frequent gains, but not necessarily playing on decks with higher long-term gains (Haines et al., 2018). Scores on the BAS Drive subscale reflect persistence in pursuing rewarding outcomes (Carver & White, 1994). Thus, individuals who pursue outcomes based on factors other than win frequency (e.g., long-term average gain) may be more persistent in obtaining rewarding outcomes.

Considering the findings from the traditional scoring and computational approaches together, our results show some relations between IGT performance and self-report measures that are consistent with previous research, but our findings also show that further work is necessary to understand how IGT performance relates to reward/punishment sensitivity and internalizing symptoms. One potential approach to improve assessment of these relations could be to estimate correlations between ORL parameters and self-report measures within a joint modeling framework, similar to how we estimated correlations for test-retest reliability. Extending the joint ORL model in this fashion was beyond the scope of our goals but is a logical next step in adapting the ORL model for specifically examining associations with self-report measures in larger samples which would allow for estimating latent constructs from the self-reports with high enough precision to capture even small effects. Regardless of the approach, further understanding the relation between IGT performance and self-reported measures of reward/punishment sensitivity and internalizing symptoms will be important for using the IGT to understand affective processing at the neural level. For example, lesion studies, in general, consistently show that the VMPFC and amygdala are involved in performance on the IGT (Aram et al., 2019), but there are inconsistencies between studies using functional neuroimaging techniques (Lin et al., 2008). An advantage of the play-or-pass IGT is that measures of reward and punishment learning are dissociable which could clarify some of these inconsistencies. Thus, an important future direction will be to examine how differences in reward and punishment learning on the play-or-pass IGT are detectable at the behavioral and neural levels within-subjects.

Although our results show promise in validating the play-or-pass IGT, these findings should be considered in the context of the study limitations. First, our sample size was modest, with 20% attrition between session 1 (*N* = 49) and session 2 (*N* = 39). Although estimates of reliability for the ORL model parameters were moderately strong, some parameters showed a high degree of uncertainty (e.g., the wide 95% credible intervals for *A*+) that could be due to a small sample. Similarly, correlations between IGT behavioral measures and self-report measures should be interpreted cautiously as the lack of a “significant” correlation could also reflect inadequate power. This aspect of the study should be considered exploratory. Future research employing larger samples may be better equipped to examine how individual differences in IGT measures relate to self-report measures. Furthermore, these studies should also sample outside of the university setting so as to maximize individual differences, particularly with respect to measures of psychopathology (e.g., the limited variability in SHAPS scores; see supplement). Another limitation that could have affected our results is that participants completed the same IGT task across both sessions. This could artificially increase reliability because participants may have remembered their choices on the previous session and made similar choices as before. Conversely, this could artificially decrease reliability if participants remembered which decks were good versus bad and specifically adjusted behavior accordingly. Despite these possibilities, we expect that any artificial increase/decrease in reliability is likely small because participants completed the tasks with a 1-month interval between assessments. Finally, our computational modeling approach focused on fitting the ORL model; however, there are alternative computational models for the IGT (e.g., the Prospect Valence Learning model; Ahn et al., 2016; Haines et al., 2018). We chose the ORL model because we previously extended this model to specifically assess test-retest reliability for the traditional IGT using a joint computational modeling framework and this approach was successful in providing reliable estimates of IGT performance (Sullivan-Toole et al., 2022). Across both studies, we have found that the ORL model provides reliable estimates of IGT task behavior; however, future studies could examine whether alternative computational models provide stronger estimates of reliability for the play-or-pass IGT.

Overall, our results show that behavior on the play-or-pass IGT, characterized by using either a traditional scoring or a computational modeling approach, is reliable across sessions and that performance on the play-or-pass IGT is related to some measures of self-reported internalizing symptoms. The computational modeling approach provided more detail regarding performance on the task because it allowed us to model trial-by-trial choices, unlike the traditional scoring approach. In addition, measures from the computational modeling approach were more widely related to self-reported measures of internalizing symptoms. These findings indicate that the computational modeling approach may be better-suited for assessing performance on the IGT and how that performance is related to other individual differences (e.g., fun seeking). At the same time, however, employing the traditional scoring approach in conjunction with the computational modeling approach allows us to understand behavior across multiple levels. At the highest level, choice proportions can be helpful in understanding how people generally behaved on the task; for example, did participants generally identify which decks were bad in the long-run and which decks were good in the long-run? At lower levels, the ORL model parameters can be helpful in understanding within-session learning that occurs during the task. Combined, both approaches may be able to capture individual differences related to decision-making deficits that are relevant for psychopathology.

## Supplementary Information

Below is the link to the electronic supplementary material.

Supplementary file1 (DOCX 7412 KB)

## Footnotes

1 Parameters from the ORL model are estimated based on data across both advantageous and disadvantageous decks, unlike traditional scoring procedures which involve calculating measures for advantageous and disadvantageous decks separately.

2 For *x*(*t*) > 0, *sgn*(*x*(*t*)) = +1; for *x*(*t*) = 0, *sgn*(*x*(*t*)) = 0; for *x*(*t*) < 0, *sgn*(*x*(*t*)) = −1.

3 We also fit the ORL model to each session individually and then assessed reliability post-hoc (i.e., a two-stage approach). As in previous research (e.g., Sullivan-Toole et al., 2022), this showed poorer reliability than the joint modeling approach. For brevity, we display the results from the two-stage approach in the supplement.
