# Supplementary material for: Test-retest reliability of the play-or-pass version of the Iowa Gambling Task
Source: Cogn Affect Behav Neurosci. 2024 Jun 7;24(4):740–54. doi: 10.3758/s13415-024-01197-6 (PMC11636993; doi:10.3758/s13415-024-01197-6)
Supplement: Supplementary file 1 — Supplementary file1 (DOCX 7412 KB) [file 13415_2024_1197_MOESM1_ESM.docx]

**Supplemental Materials**

**Supplemental Information for Self-Report Measures**

| **Table S1**  *Descriptive Statistics for Self-Report Measures* | | |
| --- | --- | --- |
| Self-Report | Session 1  *M* (*SD*) | Session 2  *M* (*SD*) |
| BAS Total | 42.89 (4.99) | 42.57 (4.31) |
| BAS Drive | 12.07 (2.31) | 11.60 (2.26) |
| BAS Fun | 12.84 (2.00) | 13.03 (1.93) |
| BAS Reward Sensitivity | 17.97 (2.06) | 17.92 (1.68) |
| BIS Total | 21.44 (3.34) | 21.19 (3.30) |
| PANAS PA | 28.84 (8.87) | 23.47 (8.66) |
| PANAS NA | 15.57 (5.96) | 13.85 (3.18) |
| MASQ General Distress Anxiety | 18.56 (7.11) |  |
| MASQ General Distress Depression | 24.82 (8.35) |  |
| MASQ Anxious Arousal | 22.44 (10.24) |  |
| MASQ Anhedonic Depression | 28.96 (15.51) |  |
| SHAPS | 13.18 (1.62) | 12.79 (1.73) |
| PROMIS-D | 28.57 (22.64) |  |

In the main text, we present correlations between the self-report measures and IGT measures in which scores on all measures were averaged across sessions prior to calculating the correlations. Below, we present correlations between IGT measures and self-reports for session 1 (Table S2) and session 2 (Table S3).

| **Table S2**  *Correlations between Self-Report Scores and IGT Measures During Session 1* | | | | | | | |
| --- | --- | --- | --- | --- | --- | --- | --- |
|  | Proportion of Plays | |  | ORL Parameters | | | |
| Self-Report | Good Decks | Bad Decks |  | *A+* | *A-* | *βf* | *βb* |
| BAS Total | -.17 [-.41,.12] | .09 [-.22,.36] |  | .19 [-.06,.39] | .05 [-.17,.28] | -.08 [-.29,.12] | -.02 [-.21,.18] |
| BAS Drive | -.25 [-.51,.06] | .12 [-.16,.43] |  | **.22 [.00,.43]** | -.05 [-.27,.16] | -.18 [-.40,.03] | -.14 [-.33,.06] |
| BAS Fun | .04 [-.25,.33] | -.11 [-.46,.23] |  | -.02 [-.29,.22] | .24 [-.01,.42] | .02 [-.17,.20] | .14 [-.12,.35] |
| BAS Reward Sensitivity | -.15 [-.38,.09] | .19 [-.06,.43] |  | **.24 [.08,.38]** | -.04 [-.25,.19] | -.01 [-.22,.21] | -.01 [-.16,.15] |
| BIS Total | .21 [-.10,.48] | -.09 [-.33,.19] |  | **-.27 [-.45,-.10]** | .15 [-.08,.34] | .15 [-.04,.33] | **.28 [.08,.45]** |
| PANAS PA | -.09 [-.35,.21] | .21 [-.20,.49] |  | .16 [-.02,.34] | .06 [-.14,.24] | **-.28 [-.44,-.11]** | .05 [-.18,.27] |
| PANAS NA | -.13 [-.38,.12] | **-.32 [-.55,-.08]** |  | **-.24 [-.40,-.10]** | .23 [-.03,.53] | -.12 [-.30,.05] | -.07 [-.25,.09] |
| MASQ General Distress Anxiety | .11 [-.14,.37] | -.25 [-.49,.04] |  | **-.23 [-.38,-.06]** | **.27 [.02,.50]** | -.01 [-.22,.17] | .14 [-.02,.30] |
| MASQ General Distress Depression | .05 [-.18,.30] | -.16 [-.44,.10] |  | -.08 [-.23,.06] | .16 [-.08,.45] | -.14 [-.35,.08] | .10 [-.03,.24] |
| MASQ Anxious Arousal | .11 [-.17,.39] | -.20 [-.48,.25] |  | -.12 [-.26,.01] | **.28 [.02,.51]** | .02 [-.17,.19] | .16 [-.04,.35] |
| MASQ Anhedonic Depression | .09 [-.24,.38] | -.17 [-.45,.22] |  | **-.27 [-.44,-.08]** | -.12 [-.33,.10] | .07 [-.10,.24] | .05 [-.19,.29] |
| SHAPS | -.05 [-.26,.17] | -.10 [-.41,.14] |  | -.02 [-.35,.22] | .09 [-.03,.21] | -.00 [-.22,.24] | -.01 [-.16,.15] |
| PROMIS-D | .13 [-.21,.42] | -.10 [-.38,.30] |  | **-.21 [-.38,-.04]** | -.07 [-.28,.14] | .01 [-.18,.20] | .14 [-.10,.38] |
| *Note.* Correlations with bootstrapped 95% confidence intervals. Confidence intervals that do not overlap with 0 are bolded. | | | | | | | |

| **Table S3**  *Correlations between Self-Report Scores and IGT Measures During Session 2* | | | | | | | |
| --- | --- | --- | --- | --- | --- | --- | --- |
|  | Proportion of Plays | |  | ORL Parameters | | | |
| Self-Report | Good Decks | Bad Decks |  | *A+* | *A-* | *βf* | *βb* |
| BAS Total | -.02 [-.41,.44] | -.00 [-.28,.35] |  | .06 [-.18,.30] | .06 [-.14,.26] | **-.21 [-.38,-.03]** | -.08 [-.30,.19] |
| BAS Drive | -.07 [-.55,.57] | .09 [-.27,.52] |  | .13 [-.17,.41] | -.10 [-.36,.15] | **-.29 [-.46,-.12]** | -.14 [-.41,.22] |
| BAS Fun | .18 [-.21,.52] | .05 [-.32,.40] |  | .01 [-.27,.28] | **.21 [.05,.36]** | -.08 [-.29,.17] | .13 [-.13,.36] |
| BAS Reward Sensitivity | -.19 [-.45,.11] | -.20 [-.44,.06] |  | -.05 [-.24,.13] | .04 [-.14,.24] | -.05 [-.25,.16] | -.17 [-.34,.04] |
| BIS Total | -.06 [-.50,.31] | -.06 [-.44,.37] |  | -.08 [-.33,.17] | -.10 [-.27,.08] | .14 [-.09,.35] | -.06 [-.32,.19] |
| PANAS PA | .16 [-.12,.40] | .08 [-.24,.40] |  | **.26 [.04,.46]** | .02 [-.19,.24] | .07 [-.16,.30] | .08 [-.13,.30] |
| PANAS NA | -.08 [-.40,.23] | -.14 [-.43,.19] |  | -.05 [-.23,.13] | .15 [-.06,.40] | -.09 [-.28,.08] | -.20 [-.42,.02] |
| SHAPS | -.14 [-.36,.17] | .04 [-.29,.40] |  | -.08 [-.39,.23] | .09 [-.05,.23] | **-.43 [-.59,-.24]** | -.13 [-.38,.08] |
| *Note.* Correlations with bootstrapped 95% confidence intervals. Confidence intervals that do not overlap with 0 are bolded. | | | | | | | |

**Supplemental Information for Computational Modeling Approach**

***Non-Centered ORL Model Parameterization***

In the main text, we present the centered parameterization of the person-level parameters in the ORL model for clarity; however, to increase efficiency, we implemented the model using non-centered parameterizations. For the non-centered parameterization, person-level parameters are assumed to following separate and independent, standard normal distributions:

|  | $z_{i,1}, z_{i,2}$ ~ $Normal\left( 0,1 \right)$ | Equation S1 |
| --- | --- | --- |

where *z_i1_* and *z_i2_* are the *z*-scored person-level parameters for participant *i* on sessions 1 and 2, respectively. To obtain estimates that follow a multivariate normal distribution, we performed the following calculation:

|  | $\left[ \begin{matrix} \theta_{i1} \\ \theta_{i2} \end{matrix} \right]\boldsymbol{=}\left[ \begin{matrix} \mu_{1} \\ \mu_{2} \end{matrix} \right]+\left[ \begin{matrix} z_{i,1} \\ z_{i,2} \end{matrix} \right]\boldsymbol{L}_{\mathbf{S}_{\boldsymbol{\sigma}}}$ | Equation S2 |
| --- | --- | --- |

where *θ_i1_* and *θ_i2_* are the person-level parameters for participant *i* on sessions 1 and 2, respectively (cf. Equation 7); *μ*_1_ and *μ*_2_ are separate and independent group-level means; and $\boldsymbol{L}_{\mathbf{S}_{\boldsymbol{\sigma}}}$ is the Cholesky factor of the covariance matrix:

|  | $\boldsymbol{L}_{\mathbf{S}_{\boldsymbol{\sigma}}}\boldsymbol{=}\left[ \begin{matrix} \sigma_{\theta,1} & 0 \\ 0 & \sigma_{\theta,2} \end{matrix} \right]\boldsymbol{L}_{\mathbf{R}_{\boldsymbol{\rho}}}$ | Equation S3 |
| --- | --- | --- |

where *σ_θ_*_,1_ and *σ_θ_*_,2_ are the group-level standard deviations for sessions 1 and 2, respectively, and $\boldsymbol{L}_{\mathbf{R}_{\boldsymbol{\rho}}}$ is the Cholesky factor of the correlation matrix (**R*_θ_***) between session 1 and session 2 person-level parameters. We reconstructed the correlation matrix **R*_θ_*** by multiplying $\boldsymbol{L}_{\mathbf{R}_{\boldsymbol{\rho}}}$ by its transpose:

|  | $\mathbf{R}_{\boldsymbol{\theta}}\boldsymbol{=}\boldsymbol{L}_{\mathbf{R}_{\boldsymbol{\rho}}}\boldsymbol{L}_{\mathbf{R}_{\boldsymbol{\rho}}}^{T}$ | Equation S4 |
| --- | --- | --- |

***Two-Stage Test-Retest Reliability for ORL Model***

To illustrate the benefits of joint modeling for improving reliability, we present test-retest correlations for the modified ORL model using a two-stage approach below. This involved fitting the ORL model to each session separately (i.e., not jointly) and calculating posteriors means for each person-level parameter across both sessions. Next, we correlated person-level parameters from session 1 with those from session 2. Figure S1 shows parameters from session 2 as a function of the parameters from session 1. Although test-retest correlations are significant for three of the four parameters, all of the test-retest correlations are weaker than those reported from the joint model (see Figure 7 in the main text). Thus, the joint modeling framework allows us to obtain more reliable person-level parameter estimates from the ORL model.

**Figure S1**

*Two-Stage Test-Retest Reliability for ORL Model*


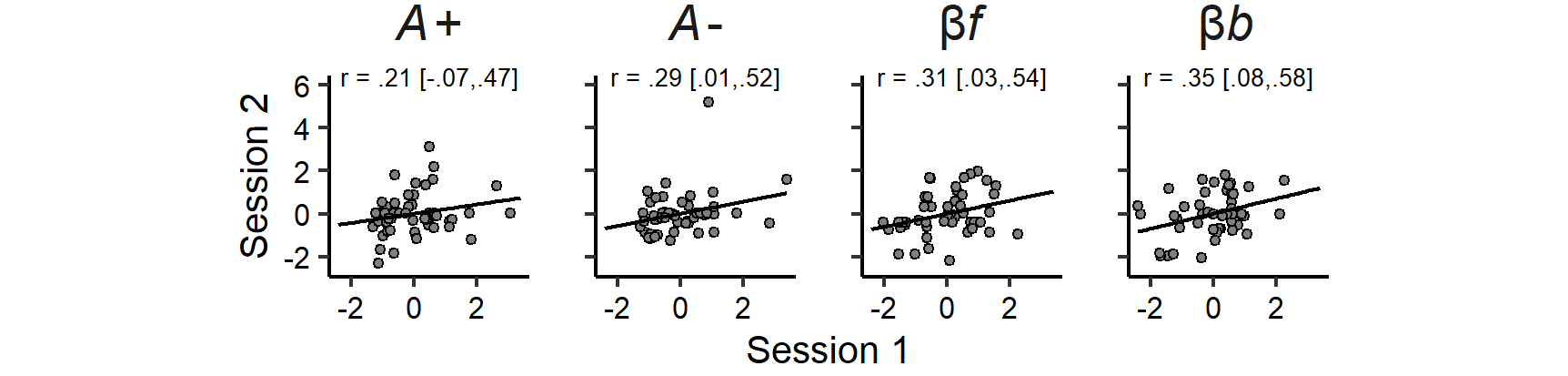


*Note.* ORL model person-level parameters during session 2 as a function of session 1. Parameters were *z*-scored for visualization purposes.

***ORL Model Parameter Recovery***

We examined whether parameters from the ORL model could be recovered to diagnose estimation issues. To do this, we simulated data from 49 participants using parameters from the fitted data. Specifically, we calculated the posterior means for each ORL parameter’s mean and standard deviation and used these means and standard deviations to sample 49 person-level parameters. For example, to obtain the person-level parameters for *βf* on sessions 1 and 2, we sampled 49 participants from the following distribution,

|  | $\left[ \begin{aligned} {\beta f}_{i,1} \\ {\beta f}_{i,2} \end{aligned} \right]$ ~ $MVNormal\left( \left[ \begin{aligned} 2.42 \\ 2.75 \end{aligned} \right] , \mathbf{S}_{\theta} \right)$ |  |
| --- | --- | --- |

|  | $\mathbf{S}_{\boldsymbol{\theta}}=\left[ \begin{matrix} 3.20 & 0 \\ 0 & 5.12 \end{matrix} \right]\mathbf{R}_{\boldsymbol{\theta}}\left[ \begin{matrix} 3.20 & 0 \\ 0 & 5.12 \end{matrix} \right]$ |  |
| --- | --- | --- |

|  | $\mathbf{R}_{\boldsymbol{\theta}}\boldsymbol{=}\left[ \begin{matrix} .59 & 0 \\ 0 & .59 \end{matrix} \right]$ |  |
| --- | --- | --- |

where *βf_i1_* and *βf_i2_* are the *βf* estimates for participant *i* on sessions 1 and 2, respectively, sampled from the multivariate normal distribution specified by the group-level posterior means of the fitted model (e.g., *M* = 2.42; see Table 1 in main text). After simulating parameters for each participant, we generated choice data for each participant and then fit the ORL model to the generated data to determine whether the simulated parameters could be recovered.

Figure S2 shows recovered parameters as a function of the actual parameters for each session. Parameters represent posterior means for each person-level parameter. Overall, we found good recovery of the parameters from the ORL model such that all correlations between recovered and actual parameters were positive and strong.

**Figure S2**

*Parameter Recovery for ORL Model*


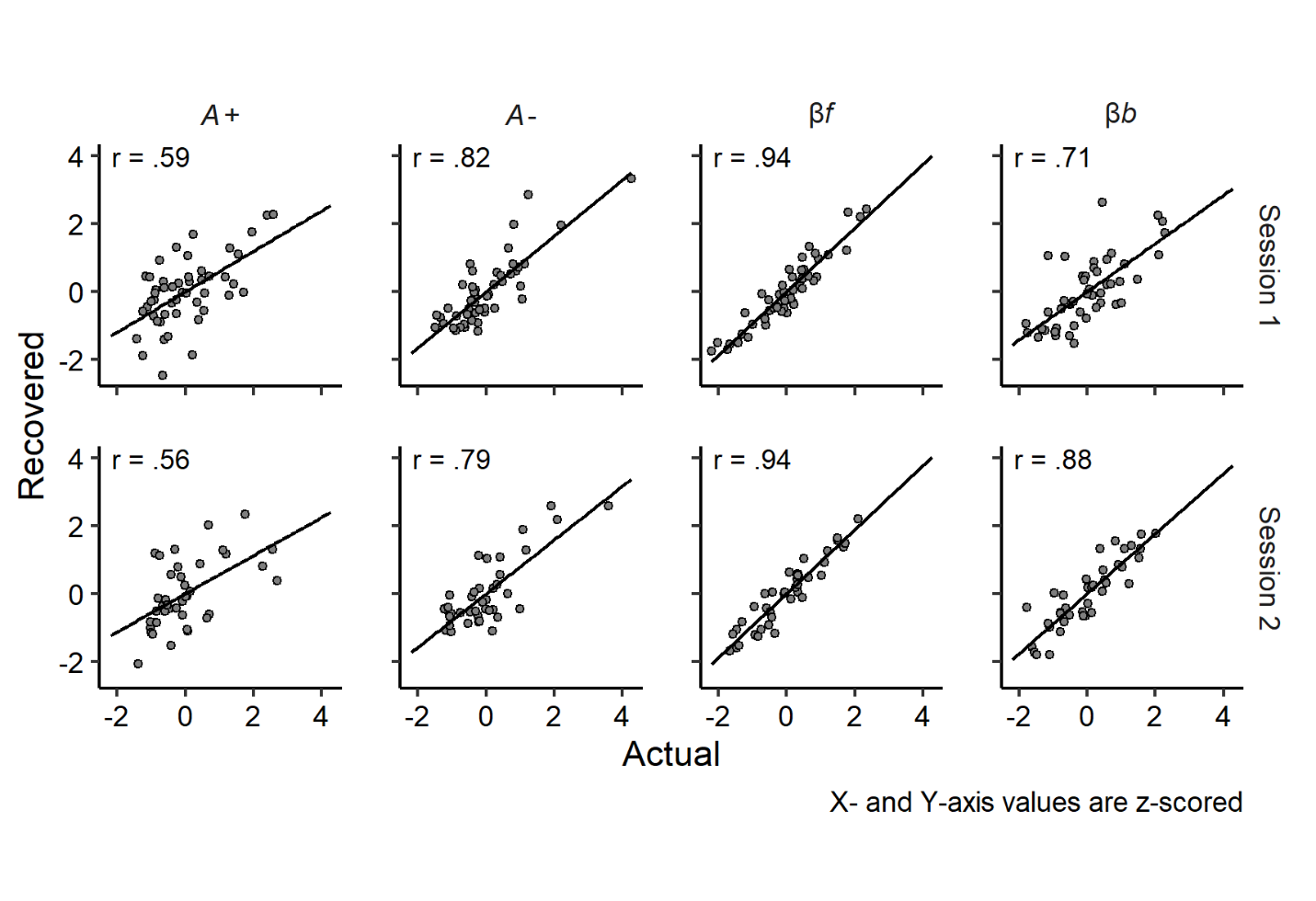


*Note.* Recovered parameters as a function of the actual parameters from the ORL model. Parameters were *z*-scored for visualization purposes.

***ORL Model Simulations***

Results from fitting the joint ORL model revealed that the group-level estimates of reward learning rate decreased, and bias increased across sessions. To illustrate the behavioral changes associated with changes in these two parameters, we simulated data from 1000 hypothetical participants using posterior means for the group-level parameter estimates from session 1 and 2. Session 1 data were simulated using session 1 posterior means for all parameters. Δ*A*+ data were simulated using the session 2 posterior mean for reward learning rate and session 1 posterior means for the other parameters, allowing us to show the behavior changes associated with decreases in reward learning rate while holding the other parameters, importantly bias, at their “baseline” levels. Similarly, Δ*βb* data were simulated using the session 2 posterior mean for bias and session 1 posterior means for the other parameters, allowing us to show the behavior changes associated with increases in bias while holding the other parameters, importantly reward learning rate, at their baseline levels. Finally, Session 2 data were simulated using session 2 posterior means for all parameters, illustrating the behavior changes associated with the combined changes in all parameters, but notably in reward learning rate and bias.

Figure S3 shows the results of the simulations which are consistent with the behavior changes described in the main text. Specifically, an increase in reward learning rate is associated with a more rapid decline in playing on a bad deck, notably Deck B, across trials within-session. A decrease in bias is associated with playing more frequently across all decks. Combined, a decrease in reward learning rate and increase in bias is associated with playing more frequently on good decks and relatively similar plays on bad decks, on average.

**Figure S3**

*Simulated Play Proportions for A+ & βb Separately & Combined*


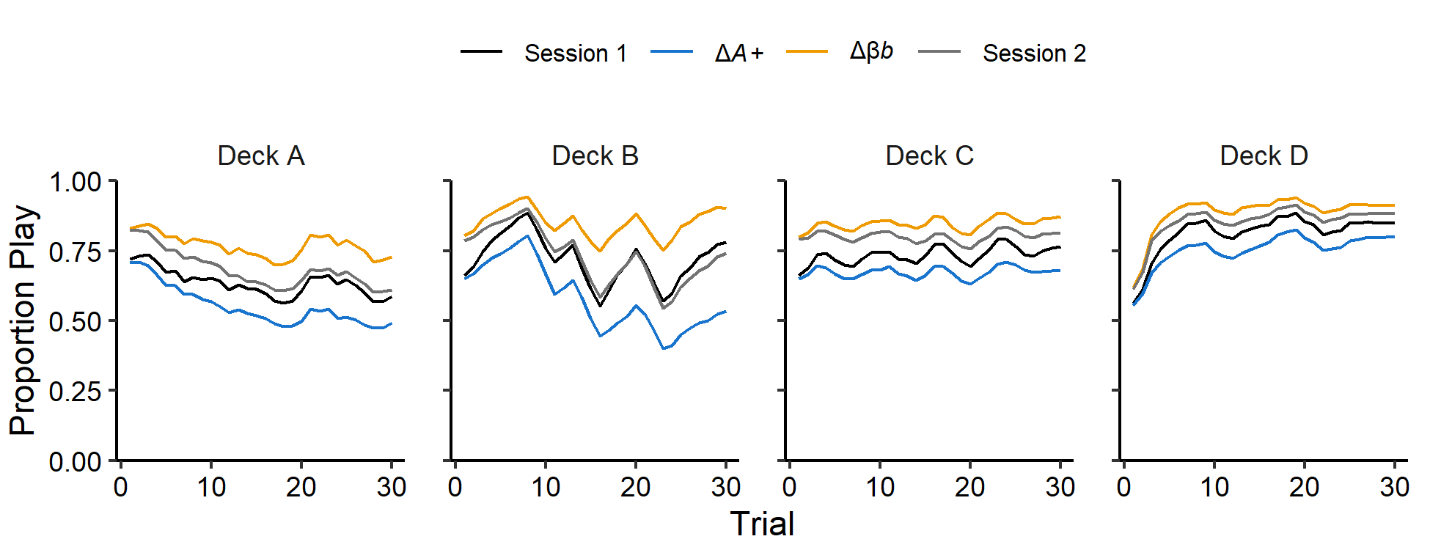


*Note.* Simulated proportion of plays across trials for each deck. To illustrate trends, data were smoothed by calculating 3-trial running averages.

***Original ORL Model***

In the main text, we present a modified version of the ORL model that does not include the perseveration and memory decay parameters from the original ORL model. We reparameterized the ORL model with a bias parameter instead of the perseveration and memory decay parameters because including those parameters resulted in poor reliability of the memory decay parameter and poor parameter recovery for the memory decay parameter and for reward learning rates (described below). In addition, we found that the perseveration parameter functioned similar to a bias parameter such that higher values of perseveration were associated with playing more frequently across most decks. Finally, model-comparisons showed that including the memory decay and perseveration parameters did not meaningfully improve model fit (also described below); thus, we present the simpler model (i.e., with four instead of five parameters) in the main text. Here, we present a summary of the results of the original ORL model as well as the model-comparisons and parameter recovery diagnostics.

**Model.** The ORL model introduced by Haines et al. (2018) and initially adapted to the play-or-pass IGT in this study included five free parameters: reward learning rate (*A*+), punishment learning rate (*A*-), win frequency sensitivity (*βf*), perseveration tendency (*βp*), and memory decay (*K*) which were used to calculate value from trial to trial using the following value-function,

|  | $V_{j}\left( t+1 \right)= {EV}_{j}\left( t+1 \right)+{EF}_{j}\left( t+1 \right)\cdot\beta f+{PS}_{j}\left( t+1 \right)\cdot\beta p$ | Equation S5 |
| --- | --- | --- |

where *V_j_*(*t* + 1) is the value of playing on deck *j* on the next trial (i.e., *t* + 1), *EV_j_*(*t* + 1) is the expected outcome value associated with playing or passing on deck *j* in the next trial, *EF_j_*(*t* + 1) is the expected win frequency of playing or passing on deck *j* in the next trial, *βf* is a free parameter describing sensitivity to win frequency, *PS_j_*(*t* + 1) is a perseverance weight that is updated from trial-to-trial, and *βp* is a free parameter describing an individual's tendency to play (when positive) or pass (when negative) on a deck based on how recently they played or passed, respectively, on that deck (operationalized as perseveration). The perseverance weight, *PS_j_*(*t* + 1), is given by the following equation:

|  | ${PS}_{j}\left( t+1 \right)=\left\{ \begin{aligned} \frac{1}{1+K} \\ \frac{{PS}_{j}(t)}{1+K} \end{aligned} \right. {if Y_{j}\left( t \right)=1 \atop otherwise}\mathrm{for} j\in1,2,3,4$ | Equation S6 |
| --- | --- | --- |

where *K* is a parameter describing how quickly participants “forget” whether they played on deck *j* across trials (further decomposed below), and *PS_j_* is the perseverance weight of each deck *j*. Note that unlike the other terms in the model, *PS_j_* is updated for all decks on each trial regardless of whether or not a play choice was made, which captures the idea that a person can forget which decks they recently “played” as a function of time. The parameter *K* is decomposed into the following equation:

|  | $K=3^{K^{'}}-1$ | Equation S7 |
| --- | --- | --- |

where *K′* is a free parameter determining the degree to which memory decays across trials. All other terms are the same as those described in the main text.

**Results.** We used the same hierarchical Bayesian analytic framework to estimate reward learning rates, punishment learning rates, win frequency sensitivity, perseveration, and memory decay in a joint model. Overall, results were similar to those described in the main text. Table S4 shows descriptive statistics and test-retest reliability estimates and Figures S4 and S5 show the mean-level and rank-order stability of ORL parameters, respectively. For the mean-level stability of parameters, reward learning rates (*A*+) decreased, and perseveration (*βp*) increased, while all other parameters remained relatively the same from session 1 to session 2 (see Table S4 & Figure S4). These findings are qualitatively similar to those of the modified ORL model. For the rank-order stability of parameters, the posterior means of the reliability coefficients for reward and punishment learning rates, win frequency sensitivity, and perseverance were moderate to strong. Of these parameters, the 95% credible interval for reward learning rate overlapped with 0 whereas the credible intervals for the other parameters did not overlap with 0. Thus, although the posterior mean of the reliability coefficient for reward learning rate was strong (*r* = .58), there was uncertainty regarding the “true” value of that reliability coefficient. Memory decay showed the poorest reliability with a posterior mean correlation coefficient close to 0 and a wide credible interval, *r* = -.09, CI [-.62, .76]. In summary, punishment learning rate, win frequency sensitivity, and perseveration showed good rank-order stability, reward learning rate showed moderate but uncertain rank-order stability, and memory decay showed poor rank-order stability.

| **Table S4**  *Descriptive Statistics & Test-Retest Reliability Estimates for Parameters from the Original ORL Model* | | | | | |
| --- | --- | --- | --- | --- | --- |
| Estimate | *A+* | *A-* | *βf* | *βp* | *K* |
| Session 1 *M* [95% CI] | .18 [.13,.23] | .10 [.08,.12] | 2.31 [1.38,3.19] | 0.86 [0.48,1.24] | 0.05 [0.02,0.08] |
| Session 2 *M* [95% CI] | .09 [.06,.12] | .09 [.07,.11] | 2.57 [1.32,3.83] | 1.68 [1.18,2.17] | 0.06 [0.03,0.86] |
| Session 2–1 *M* [95% CI] | **-.09 [-.14,-.03]** | -.01 [-.03,.02] | 0.26 [-1.01,1.54] | **0.82 [0.36,1.30]** | 0.01 [-0.04,0.05] |
| *r* [95% CI] | .58 [-.14,1.00] | **.68 [.25,1.00]** | **.63 [.32,.91]** | **.71 [.46,.93]** | -.09 [-.62, .76] |

**Figure S4**

*Absolute Stability of Parameters from Original ORL Model*


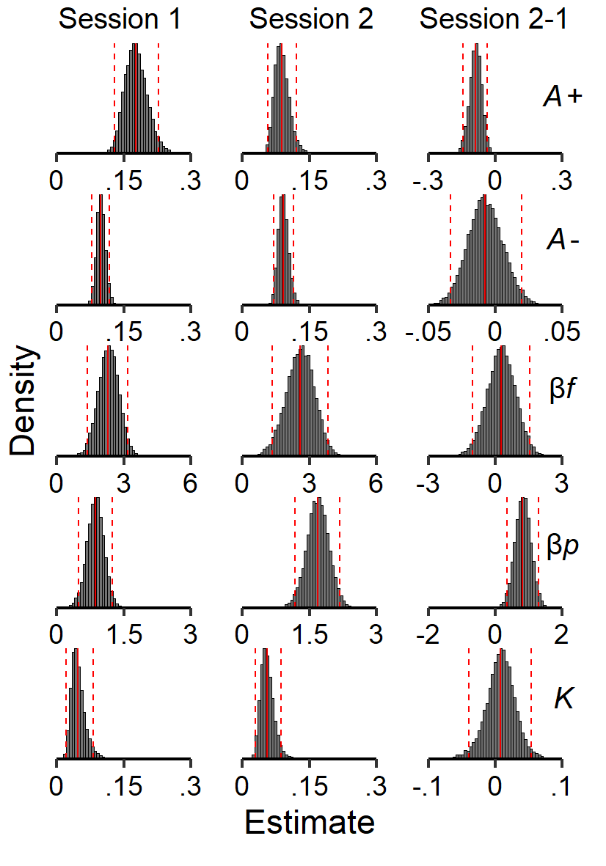


*Note.* Posterior distributions of the group-level ORL parameters from session 1 (left) and session 2 (middle), as well as the difference between session 1 and session 2 estimates (right). Solid red vertical lines represent the posterior means, and the dashed red vertical lines represent the lower and upper bounds of the 95% credible intervals.

**Figure S5**

*Relative Stability of Parameters from Original ORL Model*


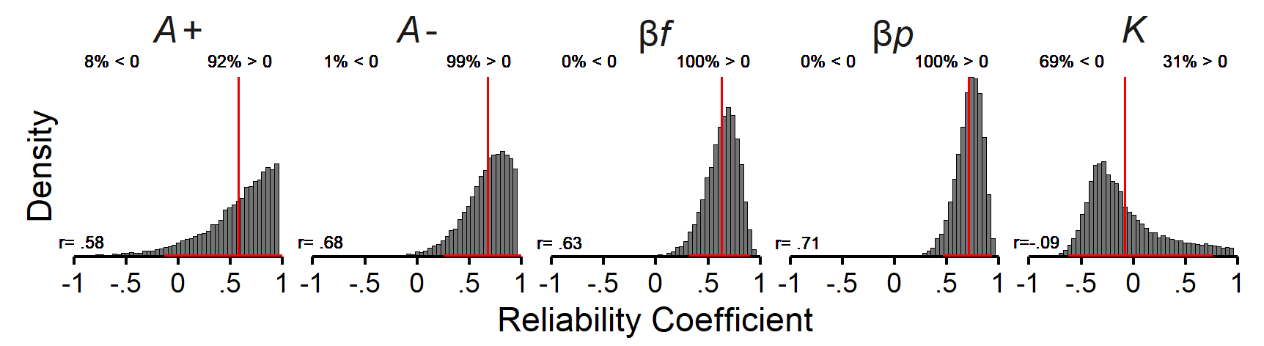


*Note.* Posterior distributions of the reliability coefficients estimated for each parameter in the joint ORL model. Solid red vertical lines represent the posterior means, horizontal lines represent the lower and upper bounds of the 95% credible intervals, and values to the left and rights sides of each panel represent the % of reliability estimates below and above 0, respectively.

**Model Comparisons.** Next, we compared the modified ORL model (with *βb*) and the original ORL model (with *K* & *βp*) according to their expected log probability densities (ELPD) and leave-one-out cross-validation information criteria (LOOIC). Overall, although the comparisons tend to favor the original ORL model, the uncertainty regarding these comparisons (i.e., the standard errors) was large (see Table S5). Thus, if the original ORL model does fit the data better, it likely does so to a small degree.

| **Table S5**  *Results of ELPD & LOOIC Model-Comparisons* | | | | |
| --- | --- | --- | --- | --- |
| Model | ELPD Diff | SE Diff | LOOIC | SE LOOIC |
| Original ORL Model | 0.0 | 0.0 | 10700.1 | 477.8 |
| Modified ORL Model | -6.2 | 30.4 | 10712.4 | 483.6 |

**Parameter Recovery.** Finally, we conducted parameter recovery diagnostics for the original ORL model (with *K* & *βp*) using the same procedures as those described above. Figure S6 shows recovered parameters as a function of the actual parameters for each session. The correlations between recovered and actual parameters were strong for punishment learning rates (*A*-), win frequency sensitivity (*βf*), and perseveration (*βp)* across both sessions and for memory decay (*K*) during session 2. Correlations between recovered and actual parameters were low to moderate for reward learning rates across both sessions and for memory decay during session 1.

**Figure S6**

*Parameter Recovery for Original ORL Model*


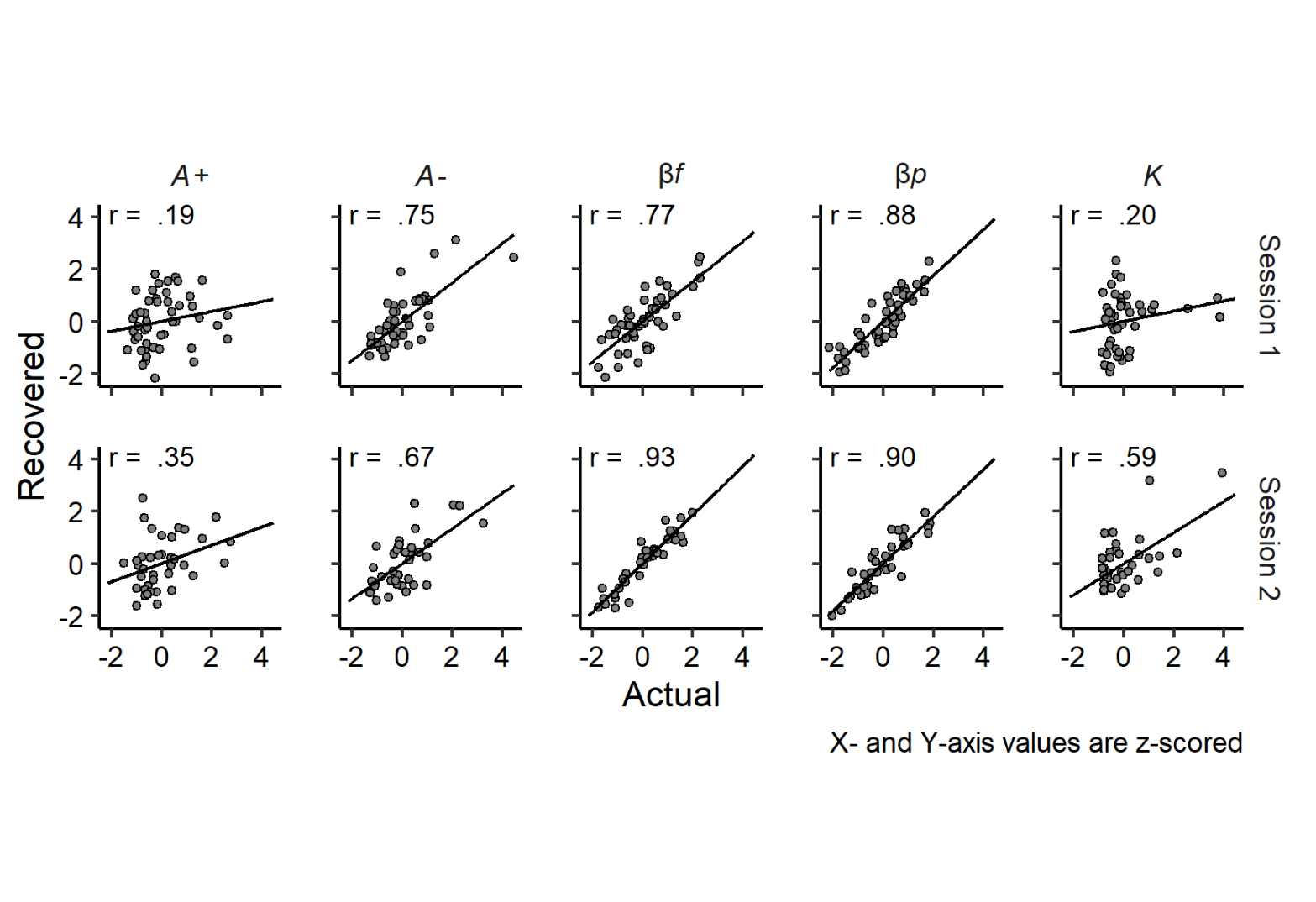


*Note.* Recovered parameters as a function of the actual parameters from the ORL model. Parameters were *z*-scored for visualization purposes.

**Person-Level PPC Plots**

**
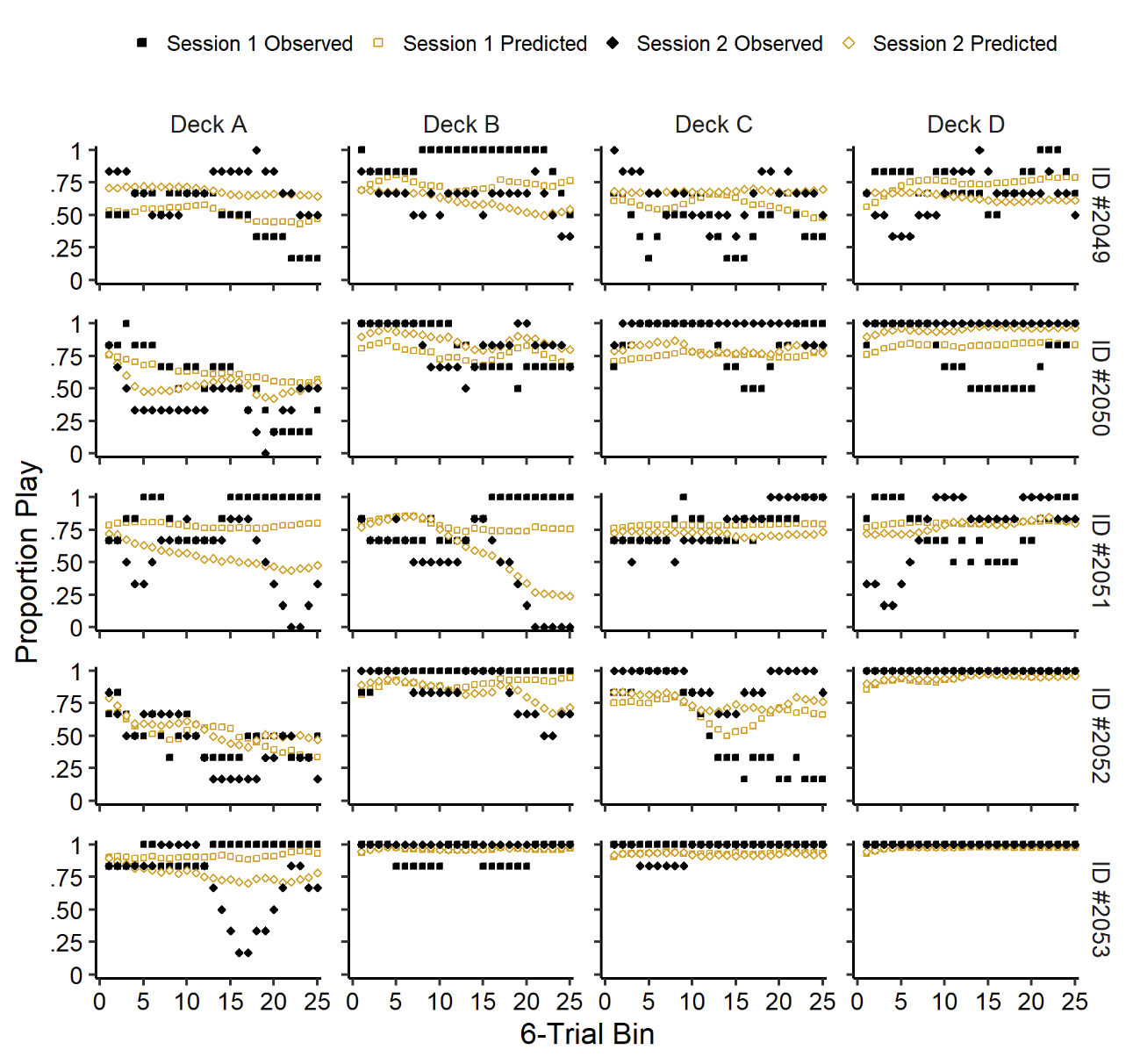

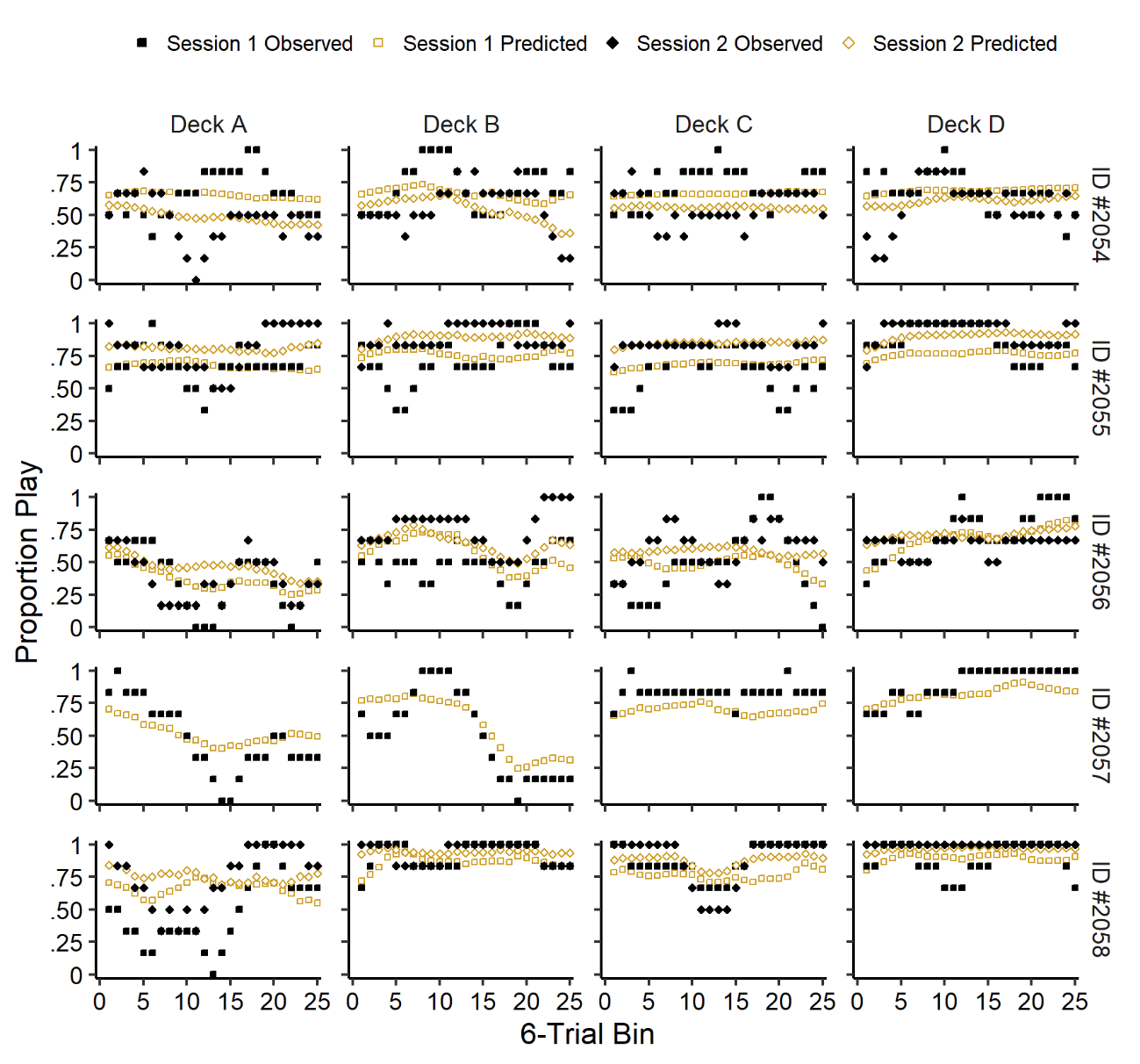

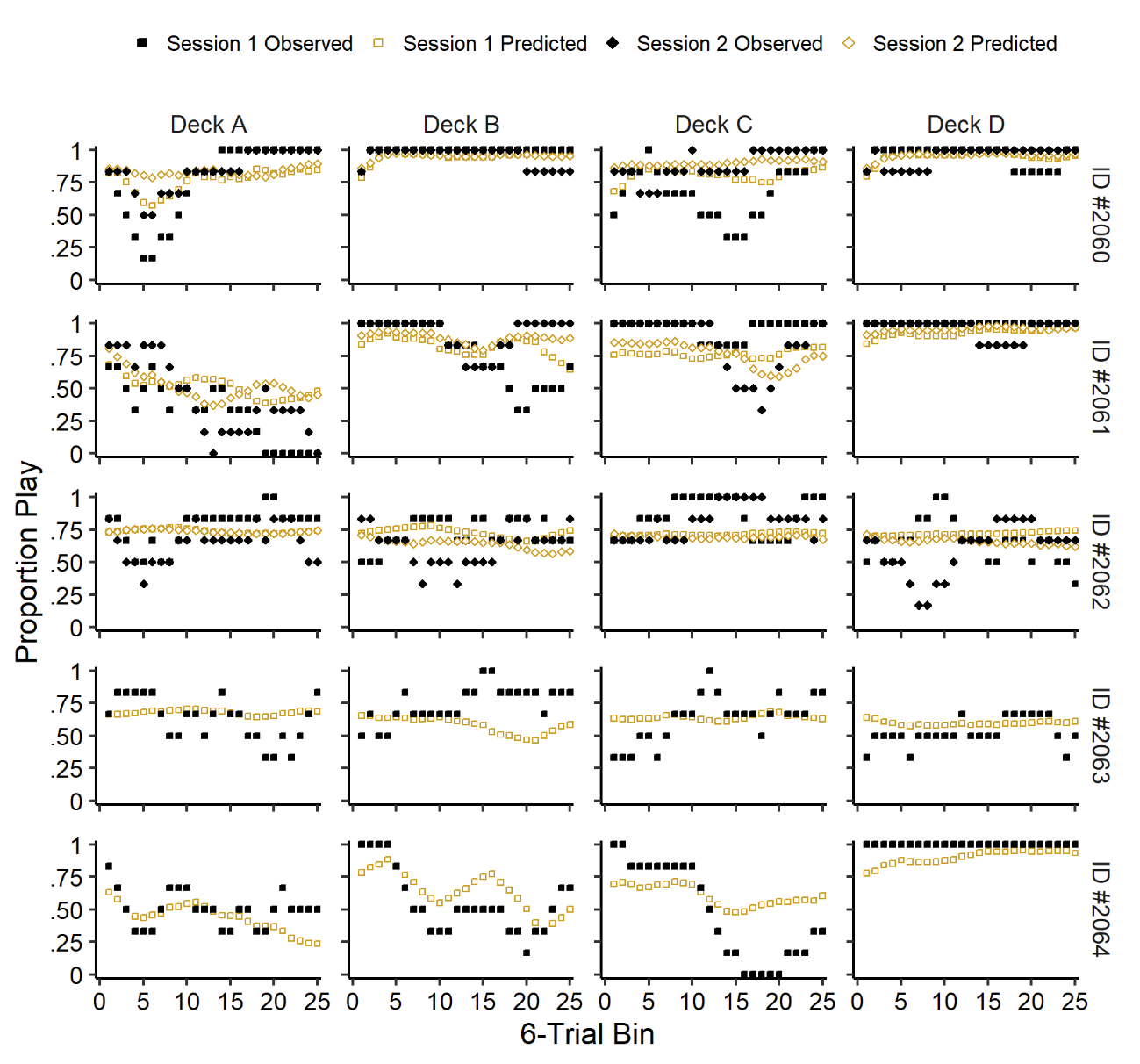

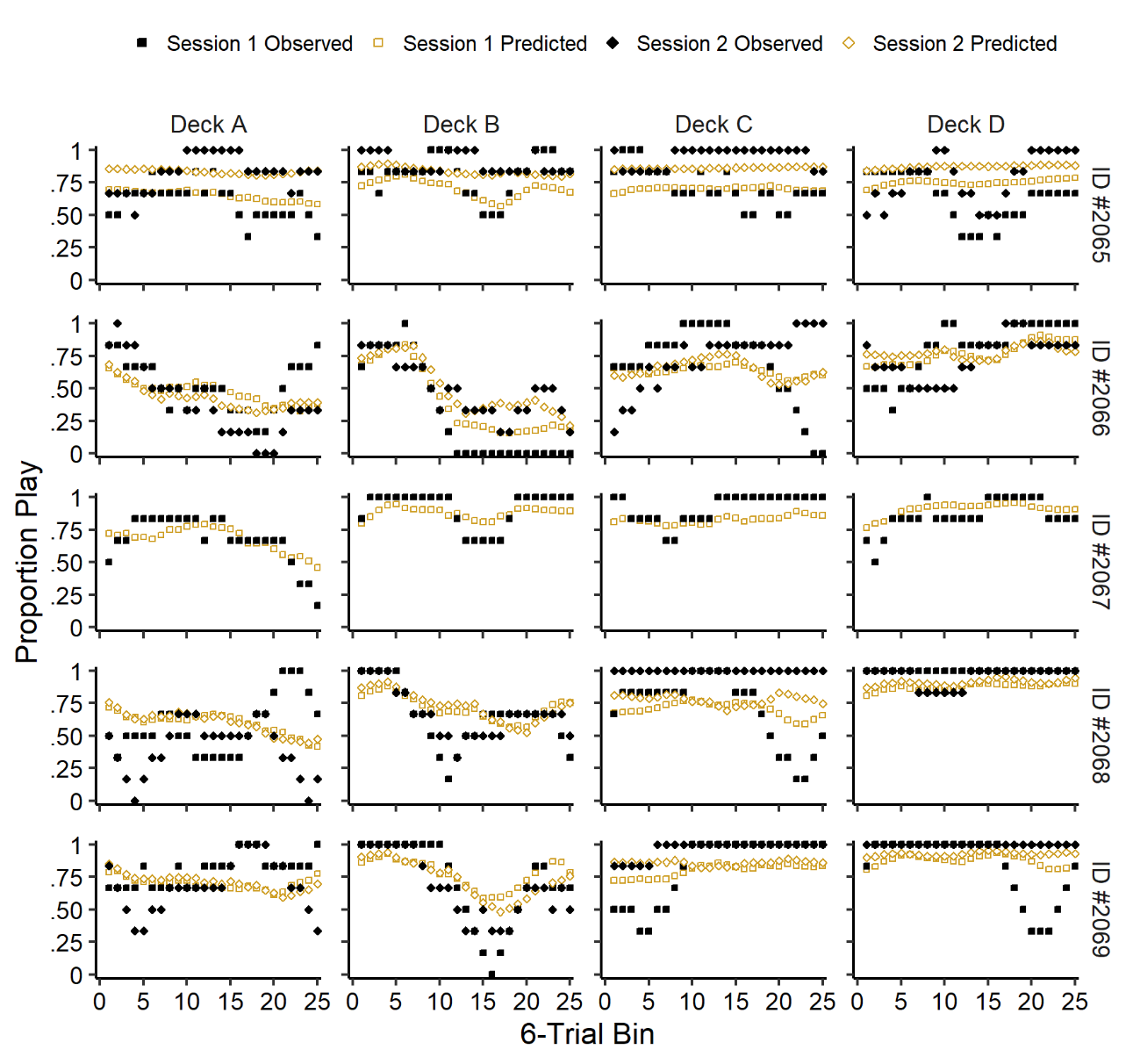

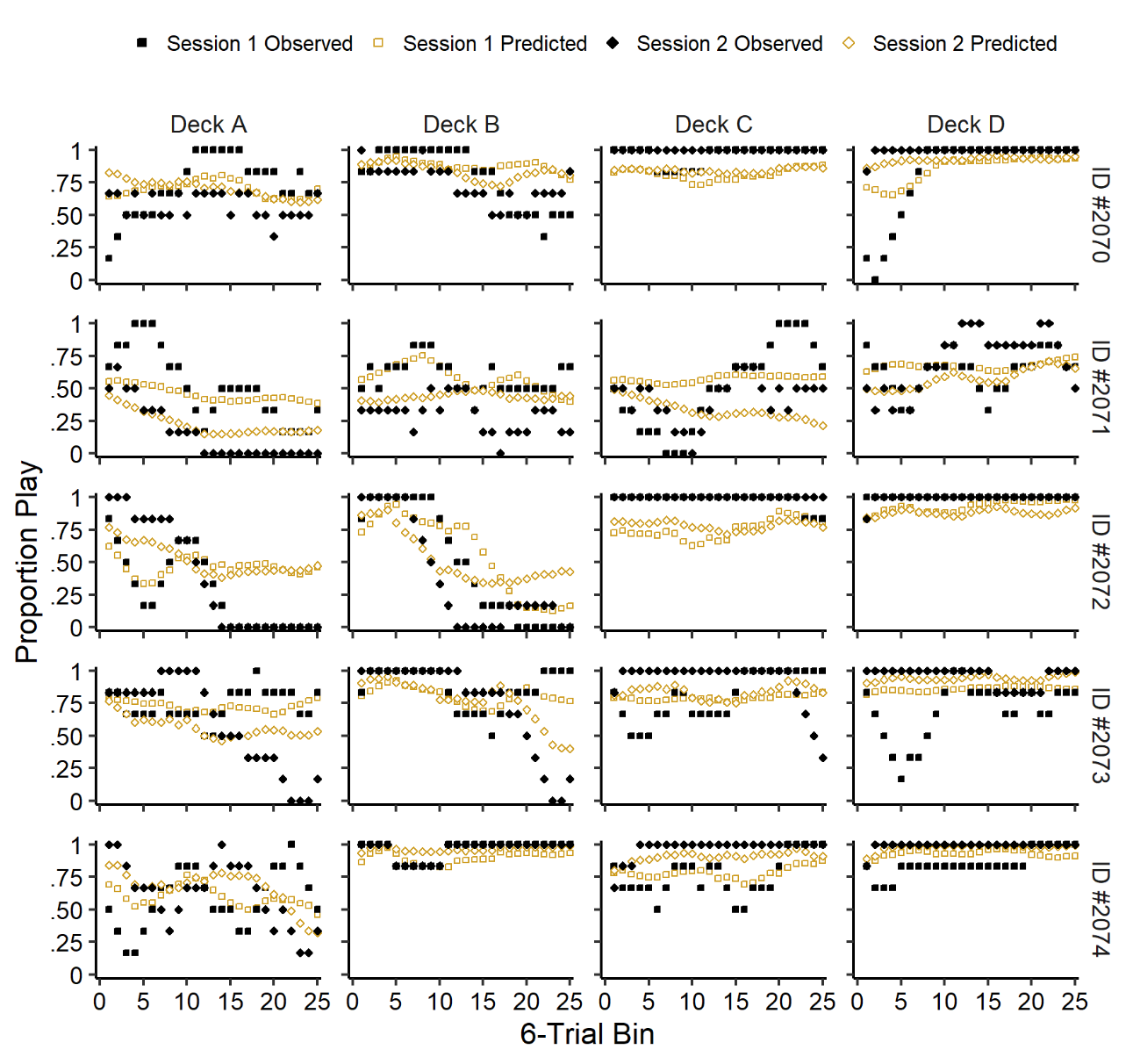

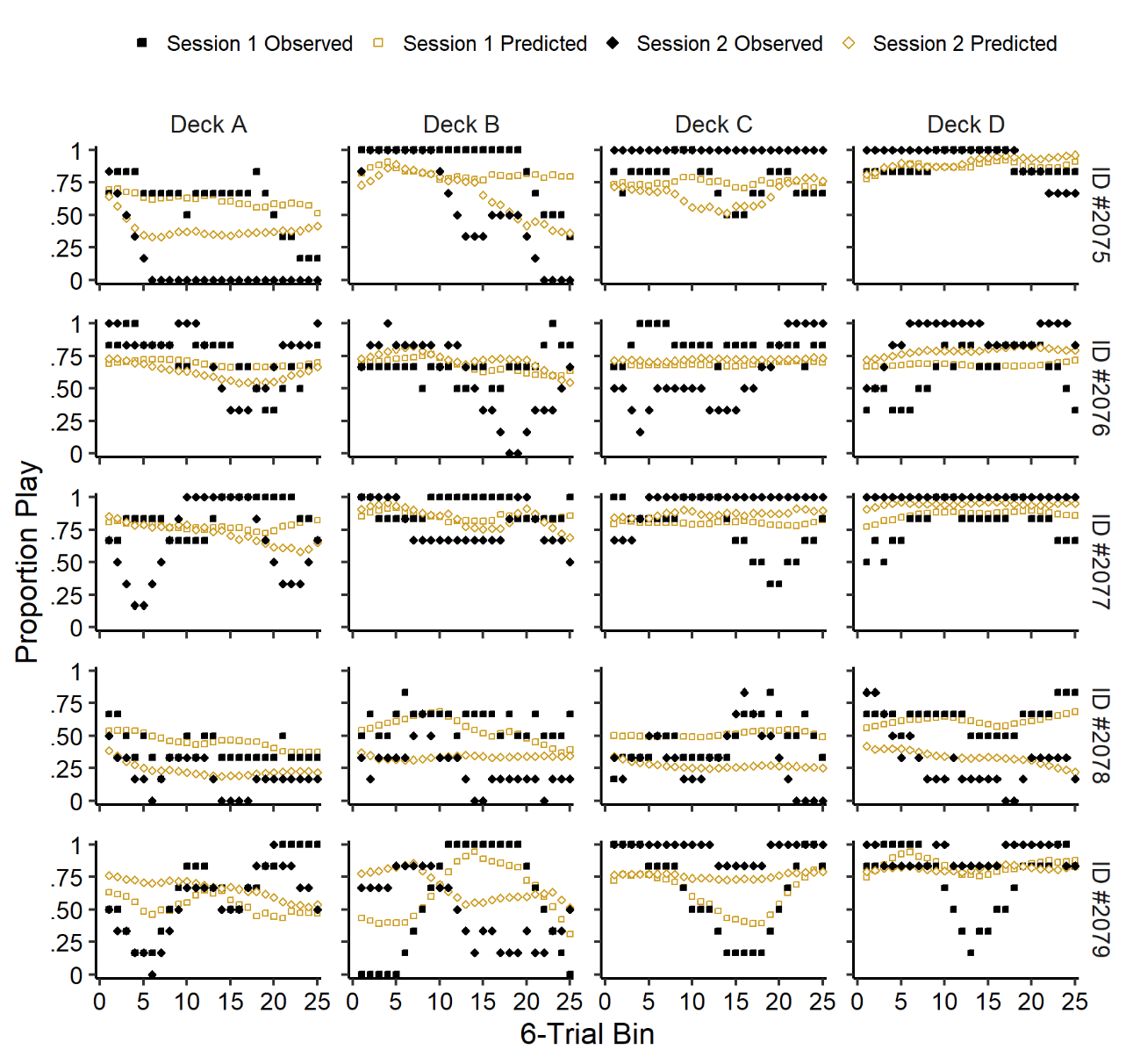

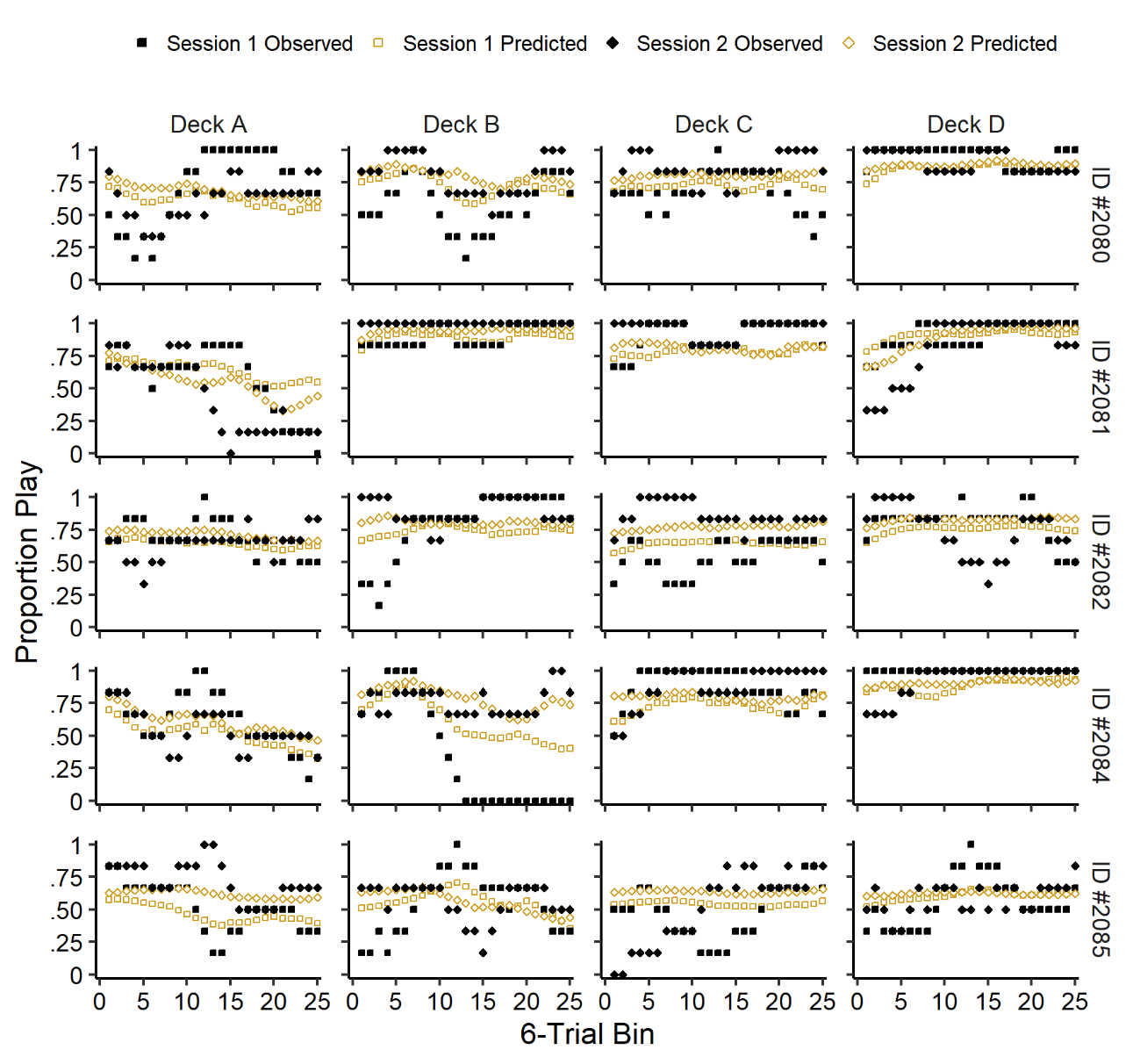

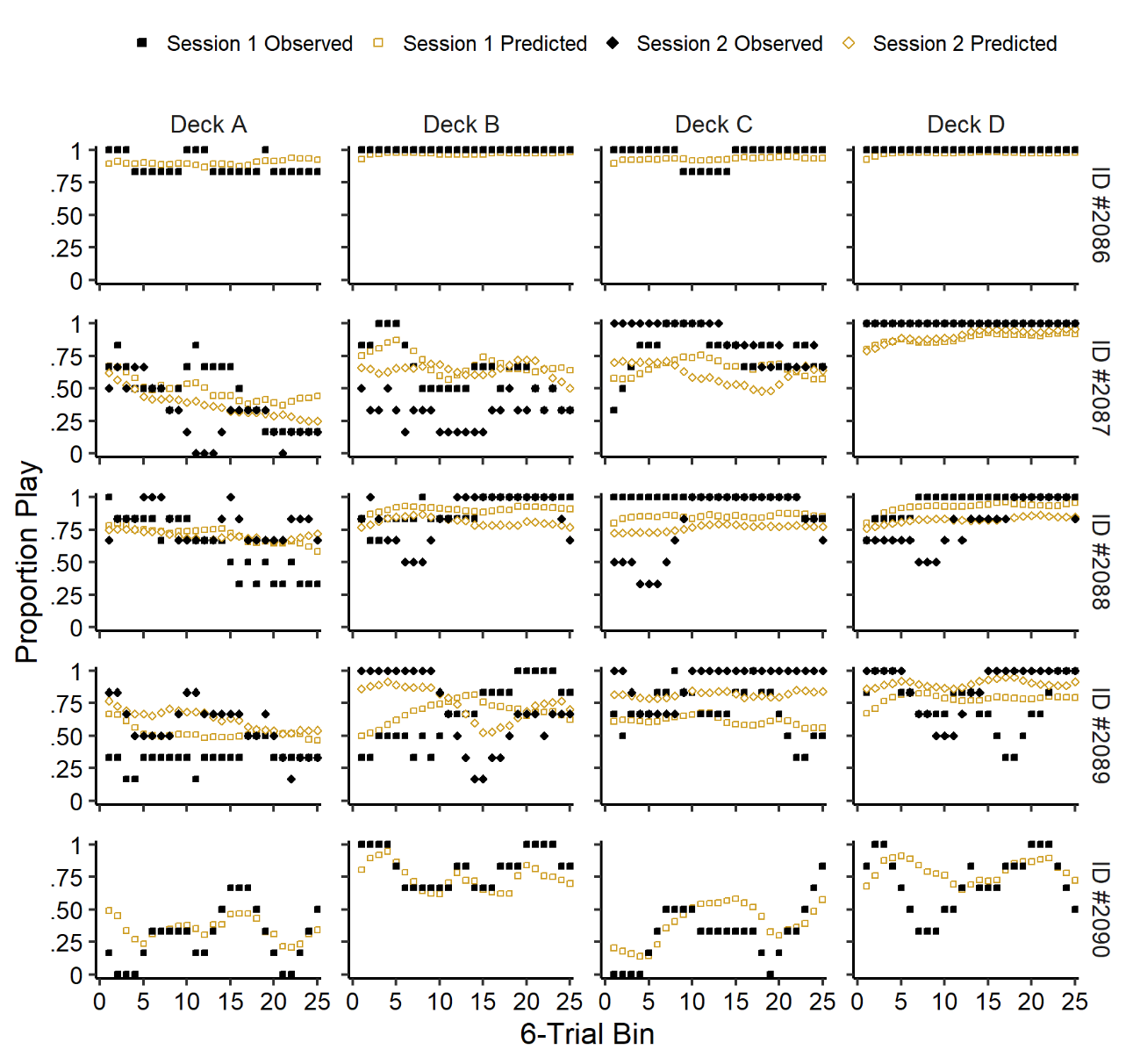

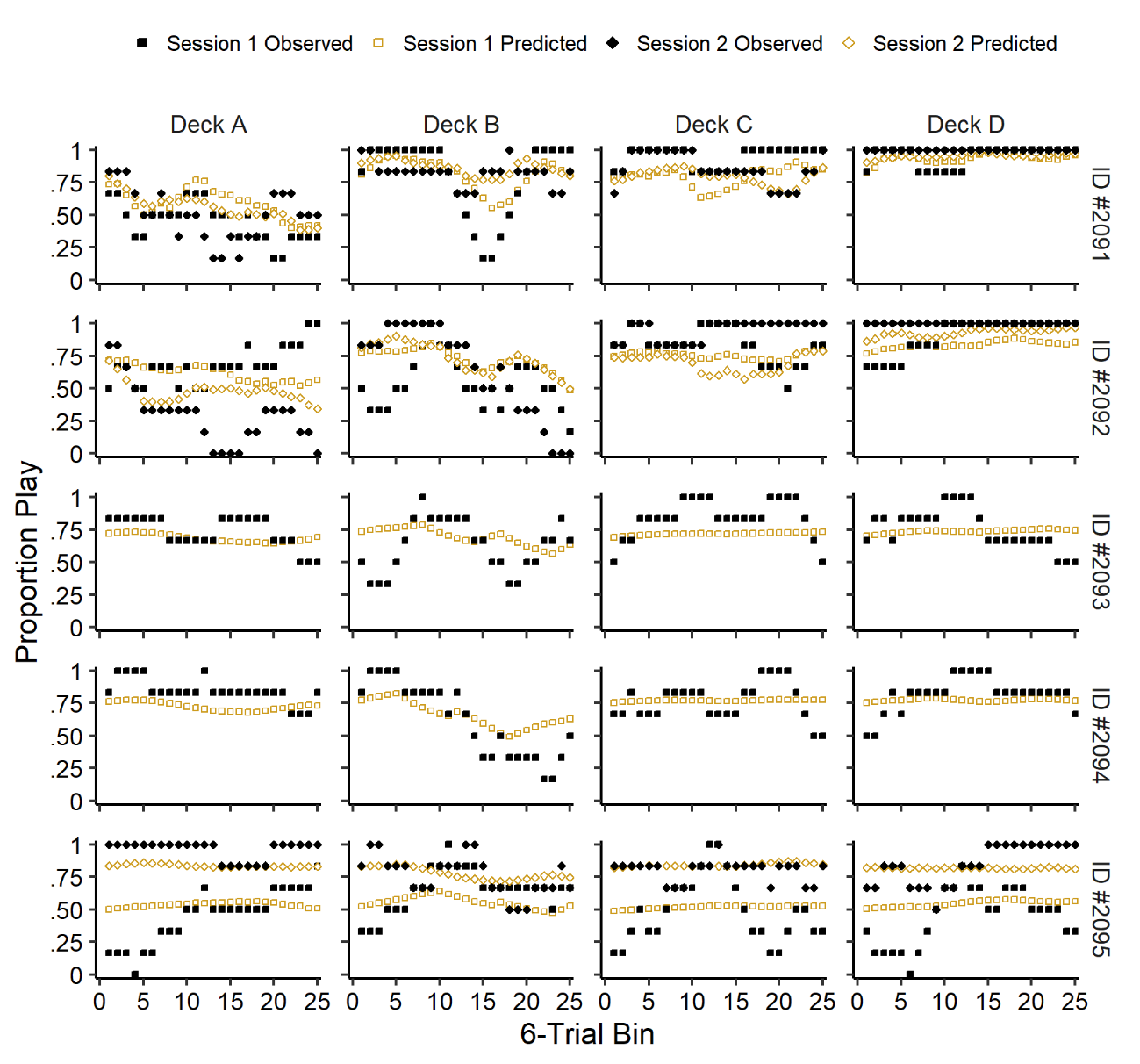

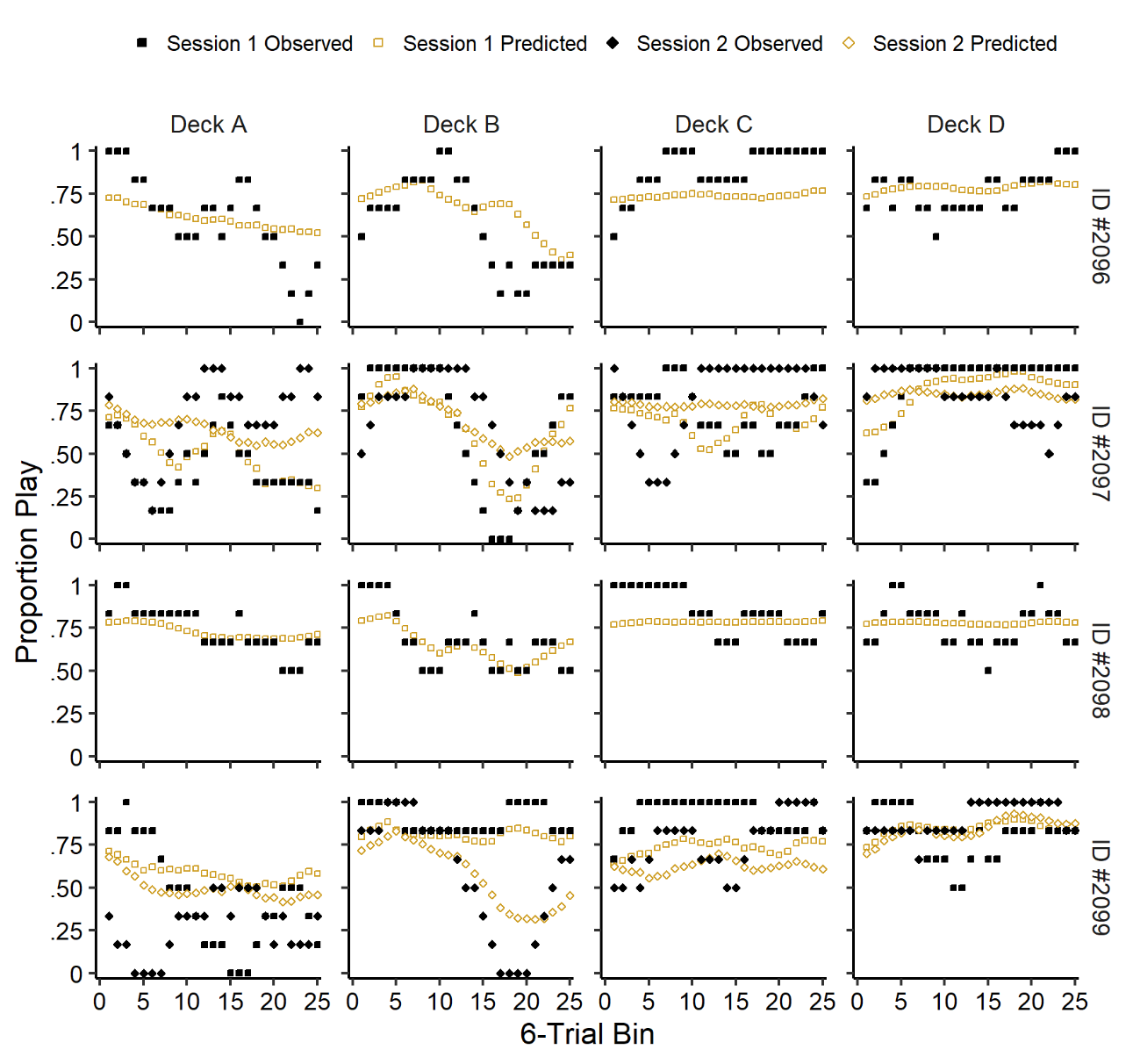
**
